# Biological mechanism of cell oxidative stress and death during short-term exposure to nano CuO

**DOI:** 10.1038/s41598-023-28958-6

**Published:** 2023-02-09

**Authors:** Elisa Moschini, Graziano Colombo, Giuseppe Chirico, Giancarlo Capitani, Isabella Dalle-Donne, Paride Mantecca

**Affiliations:** 1 Department of Earth and Environmental Sciences, Research Center POLARIS, University of Milano Bicocca, 1 Piazza Della Scienza, 20126 Milan, Italy; 2 Department of Biosciences, Università Degli Studi Di Milano, 26 Via Celoria, 20133 Milan, Italy; 3 Department of Physics, University of Milano Bicocca, 3 Piazza Della Scienza, 20126 Milan, Italy; 4 Environmental Research and Innovation (ERIN) Department, Luxembourg Institute of Science and Technology (LIST), 41, Rue du Brill, 4422 Belvaux, Luxembourg

**Keywords:** Cell biology, Nanoscience and technology

## Abstract

It is well known that copper oxide nanoparticles (CuO NPs) are heavily toxic on in vitro systems. In human alveolar epithelial cells, the mechanism of toxicity is mostly related to oxidative insults, coming from intracellularly dissolved copper ions, finally leading to apoptotic or autophagic cell death. Our hypothesis is based on possible early oxidative events coming from specific NP surface reactivity able to undermine the cell integrity and to drive cell to death, independently from Lysosomal-Enhanced Trojan Horse mechanism. Two types of CuO NPs, with different oxidative potential, were selected and tested on A549 cells for 1 h and 3 h at 10, 25, 50 and 100 µg/ml. Cells were then analyzed for viability and oxidative change of the proteome. Oxidative by-products were localized by immunocytochemistry and cell-NP interactions characterized by confocal and electron microscopy techniques. The results show that CuO NPs induced oxidative changes soon after 1 h exposure as revealed by the increase in protein carbonylation and reduced-protein-thiol oxidation. In parallel, cell viability significantly decreased, as shown by MTT assay. Such effects were higher for CuO NPs with more crystalline defects and with higher ROS production than for fully crystalline NPs. At these exposure times, although NPs efficiently interacted with cell surface and were taken up by small endocytic vesicles, no ion dissolution was visible inside the lysosomal compartment and no effects were produced by extracellularly dissolved copper ions. In conclusion, a specific NP surface-dependent oxidative cell injury was demonstrated. More detailed studies are required to understand which targets precociously react with CuO NPs, but these results introduce new paradigms for the toxicity of the metal-based NPs, beyond the Lysosomal-Enhanced Trojan horse-related mechanism, and open-up new opportunities to investigate the interactions and effects at the bio-interface for designing safer as well as more effective CuO-based biocides.

## Introduction

The nanotechnology revolution determined, and still determines, a tremendous increase in the number and type of new engineered nanomaterials (ENMs) to which humans can be potentially exposed. Thousands of commercial articles containing ENMs are already on the market, although specific safety regulations are not yet emanated by the regulatory agencies. Carbon-based NPs and metal oxides are the most produced ENMs in the world^1^ therefore the risk for humans and environment to be exposed to these compounds is quite high.

Among metal oxide nanomaterials (NMs), zinc oxide NPs and especially copper oxide (CuO) NPs have been lately investigated because of their antimicrobial properties which make them promising substitute of different toxic substances commonly used for disinfection and antimicrobial purposes. To improve their antibacterial efficacy, different synthesis techniques have been implemented. In particular, the ultrasound assisted synthesis and coating methods seem to be good candidates to obtain new antimicrobial NMs^2^. These authors hypothesized that the ability to kill bacteria depends on the generation of superoxide anions formed by the NPs in contact with microbial cells. During interaction with water, the above-mentioned metal oxides produce oxygen radicals which can finally damage nucleoids and membranes and are fatal to bacterial cells^2,3^. CuO-based NPs have also been suggested as powerful anticancer agents since their ability to exert cytotoxic effects in various tumour cell lines^4^ and to inhibit tumour growth in vitro and in vivo^5^. Moreover, it is well known that CuO NPs are highly toxic also to many non-target species, such as algae, crustaceans, fish, yeast, nematodes, protozoa, and mammalian cell lines^6^.

Almost all these studies on in vitro and in vivo systems agreed in indicating the ROS-mediated oxidative stress as the key player in the cytotoxic mechanism, but a deeper understanding of the underlying toxicity pathway and the identification of the physicochemical properties triggering this mechanism are mandatory to orient either the production of safer NMs or the development of more potent cytotoxic CuO-based nanodrugs.

Regarding the toxic behavior of CuO NPs on in vitro systems, many studies report on the very high acute toxicity in different mammalian cell lines^5,7,8^ with also attempts to find-out some correlations between the NP physicochemical properties (e. g. size, shape, solubility, surface coating) and the adverse effects observed^9–12^.

A size-dependent effect was observed in A549 cells, where nano-sized CuO particles resulted more effective than micron-sized ones in producing intracellular Reactive Oxygen Species (ROS) and genotoxic effects^13,14^.

Although metal ion dissolution can contribute to the final CuO NP-induced cytotoxicity, most papers are prone to attribute the toxic effect to the reactivity of the particles themselves. Several papers pointed-out that the contribution of the extracellularly dissolved copper ions to the CuO NP cytotoxicity accounts for less than 50% of the final effect^15,16^. The same authors agreed in indicating oxidative stress as a key mediator driving to cell death. Intracellular copper promotes the oxidative cascade inducing ROS production and glutathione (GSH) depletion, finally leading to DNA damage and cell death. However, the complete mechanism and the exact sequence of events occurring at cellular and sub-cellular levels are not completely understood.

Copper is a redox-active element able to cycle between different states (Cu^++^ and Cu^+^). The intracellular presence of both these forms coupled with the activity of intracellular enzymes participates to the production of different ROS including radical species (e. g. O_2_^°—^and OH^°^)^17^, which in turn interact with proteins, lipids, and DNA, finally leading to apoptosis or autophagy, as proposed by^2,18^.

The particles’ uptake by the so-called “Trojan horse” mechanism seems to play a key role in this pathway, since it allows particles to reach the intracellular compartments where they exert their effect through release of copper ions. By this way, the CuO NPs were seen to be internalized by endocytic vesicles, ending up in the endo-lysosomal pathway and finally engulfing secondary lysosomes where they can induce membrane leakage because of the NPs dissolution in acidic conditions, typical of the lysosomal compartment^9,19,20^.

In a previous work, our group investigated the cell-particle interactions and effects of a nanosized CuO in comparison with similarly sized TiO_2_ NPs. In that work, CuO NPs displayed very high cytotoxic effects on human epithelial lung cells (A549) exposed for up to 24 h, involving endo-lysosomal pathway activation, NP dissolution in lysosomes, significant increase of oxidative damages, and autophagic cell death^21^.

The aim of the present work is to clarify what happens at early exposure time, trying to understand if and how NP properties, such as size, crystallinity and dissolution may directly affect precocious cell responses induced by cell-NP interactions. Our tentative is to answer the following relevant questions for the nanotoxicology field: which are the CuO NP properties mainly determining the extent of the cytotoxic response? Are internalization and intracellular dissolution of CuO NPs mandatory to trigger the cytotoxic effects?

To address these issues, we compared the effects of two CuO NPs differing in primary size, crystallinity, and ROS generation capacity, by observing the bio-interactions and effects in A549 cells exposed for 1 h and 3 h. The role of oxidative stress, NP-cell interactions and endocytosis were studied by advanced molecular and morphological analyses.

## Results

### CuO NP physicochemical properties

Some of the main physicochemical characteristics of the NPs here investigated have been previously reported^2,21,22^. The measured average primary size (measured by TEM and expressed as mean ± SD) for cCuO and sCuO was 33.3 ± 10.7 nm and 24.0 ± 3.5 nm, respectively, with cCuO showing a more heterogeneous population ranging from 10 to 100 nm (see Supplementary Information file). The ROS generation capacity of the two CuO NPs, measured in a cell-free system by ESR technique, resulted significantly higher for sCuO NPs compared to cCuO^2^.

In the current work CuO NPs were further analysed by HRTEM aiming to investigate in depth the crystallite size, shape, and defects.

**Figure 1:**
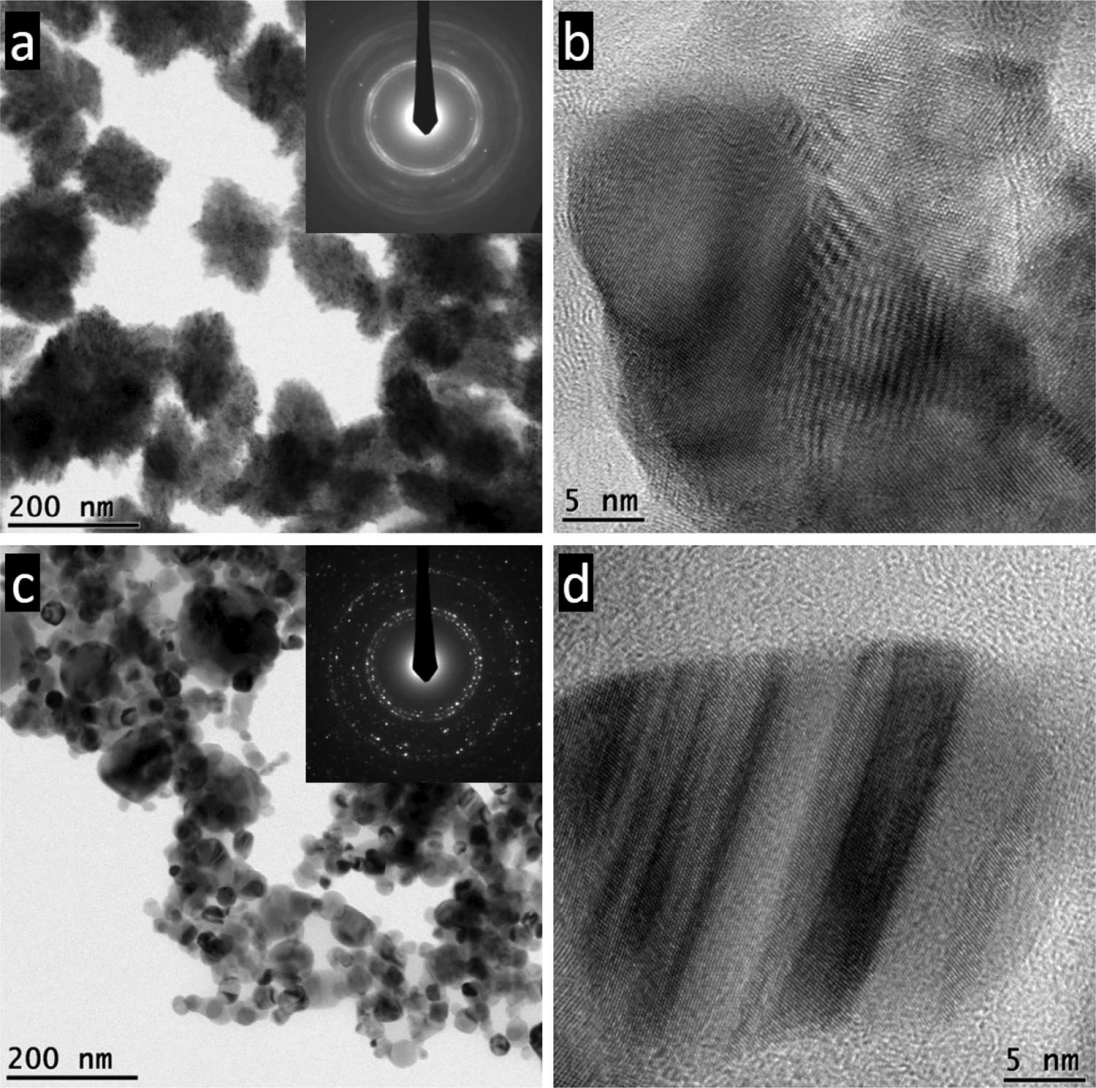
TEM images of CuO NPs. (**a**) Bright field image of sCuO NPs and related diffraction pattern (inset); (**b**) High resolution image of NPs as those reported in (**a**); (**c**) Bright field image of cCuO NPs and related diffraction pattern (inset); (**d**) High resolution image of a small nanoparticle of cCuO showing twinning bands.

The two samples appeared radically different, both morphologically and dimensionally, and appeared as aggregates, sCuO in particular. sCuO was very small in size, possibly flat, forming leaf-like clusters up to few hundreds of nm and having a spotted contrast (Fig. 1a). They are randomly oriented, as testified by the selected area diffraction pattern (Fig. 1a, inset). High resolution images show that coherent diffracting volumes have size no larger than few tens of nm in diameter and that the spotted contrast observable at low magnification is due to superposition of particles that generate largely spaced fringes by Moirè effects (Fig. 1b). cCuO forms bunches of discrete NPs with roughly spherical shapes and diameter up to one hundred of nm (Fig. 1c). The larger size of cCuO is also testified by the electron diffraction pattern (Fig. 1c, inset), where a lower number of diffraction spots can be recognized within the same diffracting area with respect to the case of sCuO (Fig. 1a). At high magnification, cCuO shows larger coherent diffracting volumes, even if often affected by parallel bands ascribable to stacking faults or twinning, as it is the case of the small NP imaged in Fig. 1d. The smaller size and the more defective/disordered crystal structure of sCuO, may thus be responsible for the higher biological reactivity due to an increased ROS generation capacity, as characterized in Perelshtein et al.^2^.

Once diluted in the cell culture medium at the selected concentrations of 25 µg/ml and 50 µg/ml at 37 °C, the CuO NP suspensions were analysed by DLS.

The analysis of the autocorrelation functions of the scattered light revealed that at least two components were necessary in Eq. 1 to fit the autocorrelation functions of the light scattered by NPs supended in cell culture medium at the test concentrations. The two components corrisponded to widely different hydrodynamic radii (see Supplementary Table 1 and Supplementary Fig. S4) and are likely due to the single NPs and to rare, large, aggregates. This hypothesis was confirmed by the maximum entropy analysis of the first order correlation functions, according to Eq. 2. The distributions of the hydrodynamic radii (see Supplementary Table 2 and Supplementary Figure S5) show typically a single peak with a smooth tail on the large size edge. The radii distributions could be described very well by the sum of four log-normal components, so that the average hydrodynamic radii could be computed, as reported in Supplementary Table 1. These values agree well with the hydrodynamic values of the fastest component detected in the autocorrelation functions by means of a double exponential decay fit, confirming that, apart from the case of cCuO (50 µg/ml), the major component present in the suspension is that of non-aggregated nanoparticles. Anyway, it is clearly shown that cCuO formed larger aggregates than sCuO once dispersed in cell culture medium.

The NPs dispersed in milliQ water displayed a positive surface charge of + 15.1 ± 9.4 mV (cCuO) and + 29.3 ± 5.3 mV (sCuO). Once diluted in culture medium, the z-potential turned to comparable negative values of -8.6 ± 0.5 mV and -9.2 ± 0.1 mV for cCuO and sCuO respectively. The reported values refer to the NP concentration of 100 µg/ml.

**Figure 2:**
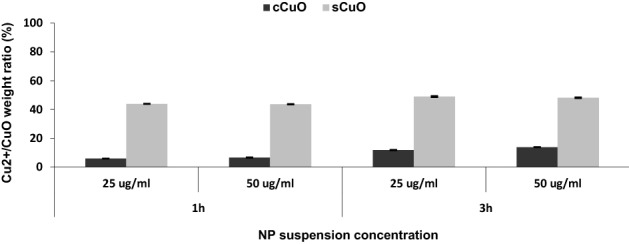
Extracellular copper ions release. The amount of copper ions released by cCuO and sCuO (25 µg/ml, 50 µg/ml) was measured by ICP-OES after 1 h and 3 h of incubation in cell culture medium. Results are shown in the graph as mean [Cu^2+^/CuO] weight ratio percentage ± SD.

ICP analysis was performed to assess the release of copper ions in culture medium during exposure. As summarized in Fig. 2, a slight time-dependent dissolution was observed for both CuO NPs (especially for cCuO), with sCuO showing higher dissolution ratios than cCuO. After 3 h of incubation, about 12% of cCuO and 48% of sCuO resulted dissolved, confirming the partial solubility of CuO NPs in cell culture medium.

### Cell viability

**Figure 3:**
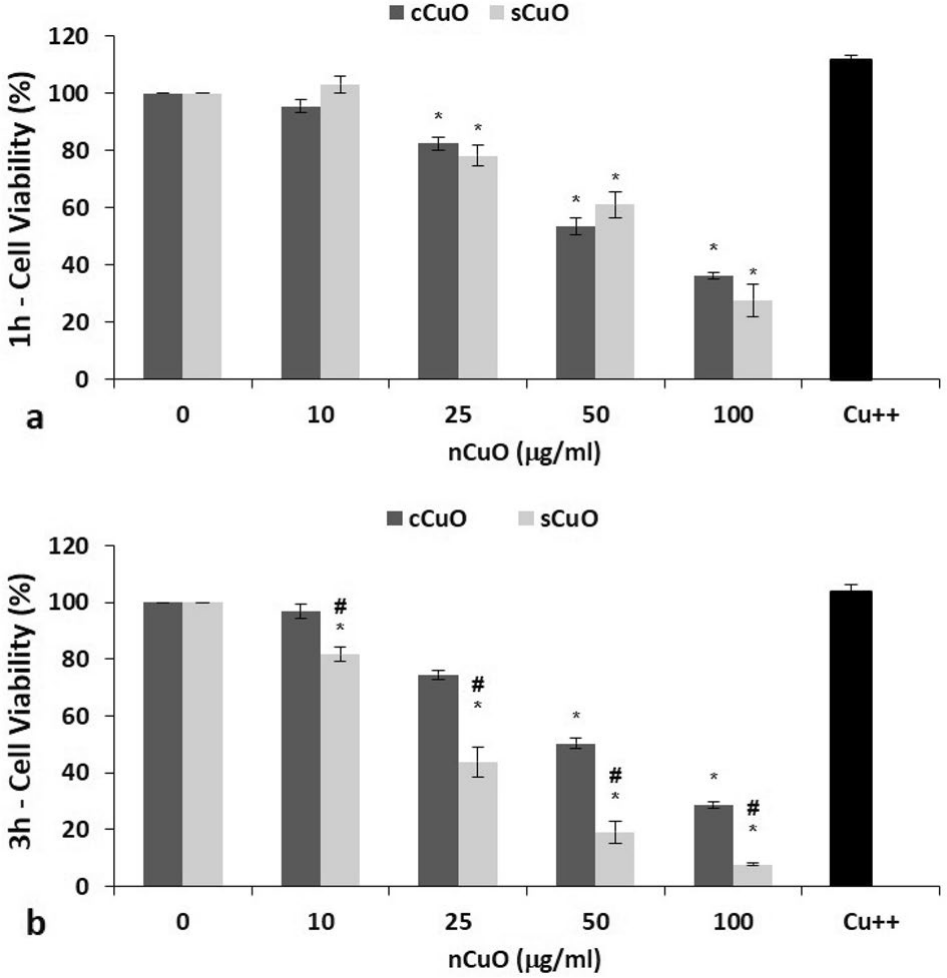
Cell viability results by MTT assay. A549 cells were exposed to cCuO and sCuO for 1 h (**a**) and 3 h (**b**). Dark grey bars = cCuO; light grey bars = sCuO; black bar = 80 µg/ml Cu^++^ (from CuSO_4_·5H_2_O). Results are expressed as mean ± SD. *Significantly different from control (ANOVA + Fisher LSD Method, p < 0,05). ^#^Significantly different from the other experimental group tested at the same concentration (t-test, p = 0,002).

In a previous work, we demonstrated that the results from the MTT assay can efficiently reflect the viability of CuO NP-exposed cells^21^. In this study, MTT underlined a concentration- and time-dependent cytotoxicity in A549 after exposure to sCuO NPs (Fig. 3a,b) while only a concentration-dependent effect was seen after exposure to cCuO. A significant decrease in cell viability was already observed starting from 25 µg/ml of cCuO and sCuO. Although after 1 h exposure the effects were comparable between cCuO and sCuO, after 3 h the viability decrease was significantly higher after cell exposure to sCuO. After 3 h exposure to 100 µg/ml sCuO, cell viability was almost null. Light microscopy images showing the morphological effects on CuO-exposed cells, and confirming the MTT results, are reported in the section "Cell-CuO NPs bio-interactions by microscopic techniques".

NPs themselves were responsible for the CuO-induced viability decrease, since no effects were produced by soluble copper tested at the maximum concentration (80 µg/ml) potentially released from the highest NP concentration (100 µg/ml) (Fig. 3a,b).

### Oxidative stress markers

#### Oxidation of protein thiols and protein carbonylation

Oxidation of protein thiols and protein carbonylation were investigated as precocious markers of reversible and irreversible oxidative damage respectively^23^. In particular, protein carbonylation was assessed by both immunoblotting and immunochemical method after derivatization with 2,4-Dinitrophenylhydrazine (DNP)^24^.

**Figure 4:**
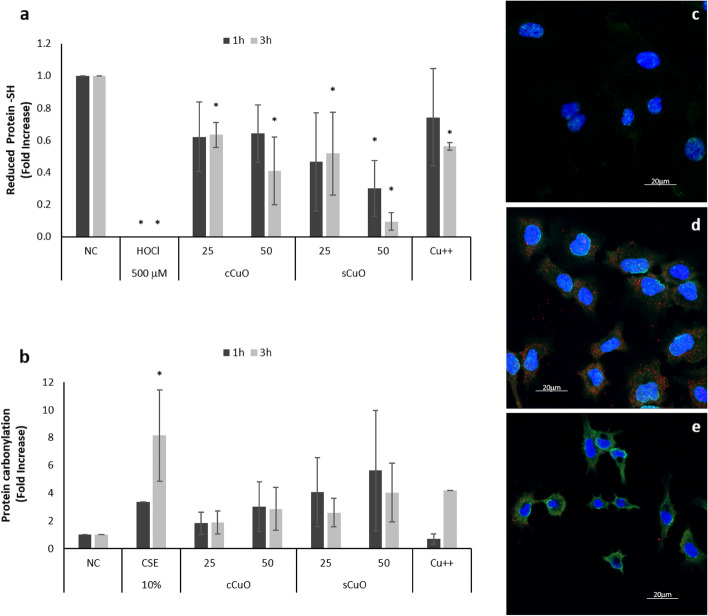
Short-term oxidative stress markers in A549. Histograms (**a**, **b**) show the results obtained by immunoblotting after the exposure of cells to cCuO and sCuO for 1 h (dark grey bars) and 3 h (light grey bars) about the quantification of (**a**) Reduced protein thiols; (**b**) Protein carbonylation (PCO). NC) Negative Control (unexposed cells); HOCl = 500 µM sodium hypochlorite; CSE = 10% condensate smoke extract; Cu^++^ = 80 µg/ml copper from CuSO_4_·5H_2_O. Results are expressed as mean ± SD. *Significantly different from control (ANOVA + Fischer LSD Method, p < 0,05). The pictures (**c**–**e**) show the immunocytochemical detection of protein carbonyls by confocal laser scanning microscopy (CLSM) after 3 h of exposure; (**c**) negative control cells; (**d**) cCuO-exposed cells; (**e**) sCuO-exposed cells. Nuclei are stained by Hoechst (blue spots); CuO NPs are mapped by reflection mode (pseudocolored red spots); protein carbonyls were immunostained with anti-DNP (green fluorescence).

As shown in Fig. 4a, a significant and concentration-dependent decrease in reduced protein thiol content was observed already after 1 h of exposure, especially to sCuO. At 100 µg/ml both cCuO and sCuO almost completely oxidized the protein thiols (data not shown), whereas at the intermediate concentrations of 25 µg/ml and 50 µg/ml the effect of the sCuO was definitely higher (p < 0,05).

Figure 4b summarizes the CuO-induced protein carbonylation in whole-cell lysates analysed by Western immunoblotting. Both NPs induced a remarkable increase, although not statistically significant, in protein carbonylation, when compared to untreated cells.

In parallel, we performed the confocal microscopy imaging of cells for DNP immunofluorescence and for NP mapping by laser reflection mode, according to^25^. This allows the qualitative evaluation of the carbonyl-specific signal at the cellular level together with the identification of cell-interacting NPs. After 3 h, the protein carbonylation signal was almost undetectable in control cells (Fig. 4c), while A549 cells exposed to cCuO NPs showed a slight DNP immunoreactivity spread throughout the cytoplasm, in association with a relatively abundant presence of scattered NPs (Fig. 4d). A strong carbonylation signal (green spots), likely coming from the cytoplasm and from the cell membrane systems, was shown in the sCuO-exposed cells, although fewer NPs seemed to be internalized or adherent to the cells (Fig. 4e).

#### Role of NAC in preventing cell viability decrease

**Figure 5:**
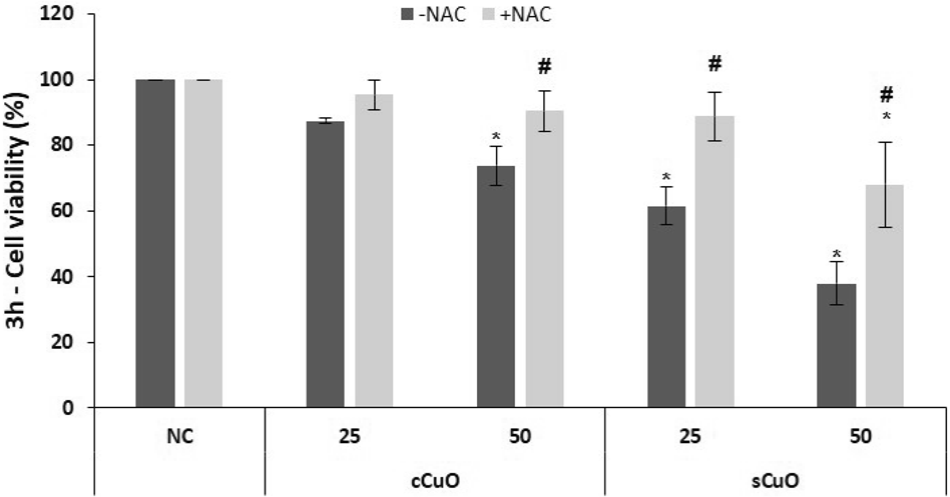
Cell viability results by MTT assay of A549 co-incubated with a ROS scavenger. Cells were exposed to copper oxide nanoparticles for 3 h in presence of 10 mM NAC (+ NAC, light grey bars) or without (-NAC, dark grey bars). Results are expressed as mean ± SD. *Significantly different from the negative control (NC) (ANOVA + Fischer LSD test; p < 0,05). ^#^Significantly different from the correspondent experimental group without NAC (t-test, p = 0,002).

By incubating cells with the antioxidant N-acetyl-cysteine (NAC), a significant viability recovery was achieved in A549 exposed to both CuO NPs (Fig. 5). The efficacy of the antioxidant treatment was very evident in the cells exposed to the most reactive sCuO, for which a very effective recovery was obtained at both 25 µg/ml and 50 µg/ml (Fig. 5). This suggests that the viability decrease is triggered by an early cell oxidative imbalance and protein thiols might be specific targets of the CuO NP oxidative potential. However, further experiments will be needed to confirm this hypothesis.

### Cell-CuO NPs bio-interactions

#### Morphological analyses

Cell-particle interactions were investigated by microscopy techniques to better understand if and how NPs were internalized and if morphological changes occurred at early exposure stages.

**Figure 6:**
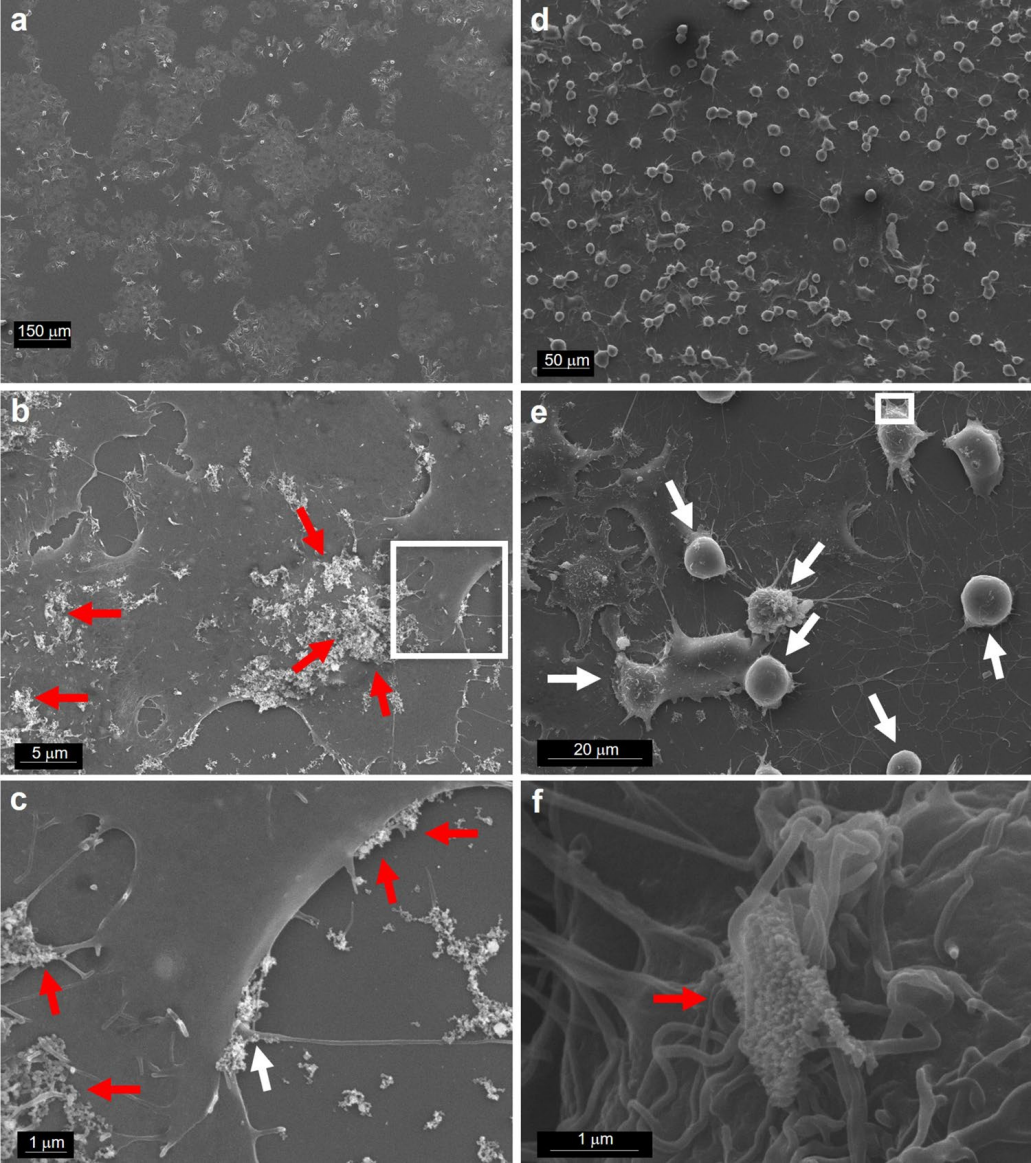
SEM images of A549 exposed for 3 h to CuO NPs. (**a**) unexposed cells; (**b**) cells exposed to cCuO at 50 μg/ml—aggregates of nanoparticle are visible on the cell surface (red arrows); (**c**) detail of (**b**)—white frame—showing cCuO interacting with the plasma membrane; (**d**, **e**) different magnifications of cells exposed to sCuO at 50 μg/ml—numerous mitotic/apoptotic cells (white arrows) are appreciable; (**f**) detail of (**e**) showing sCuO NPs agglomerate on cell surface (red arrow).

**Figure 7:**
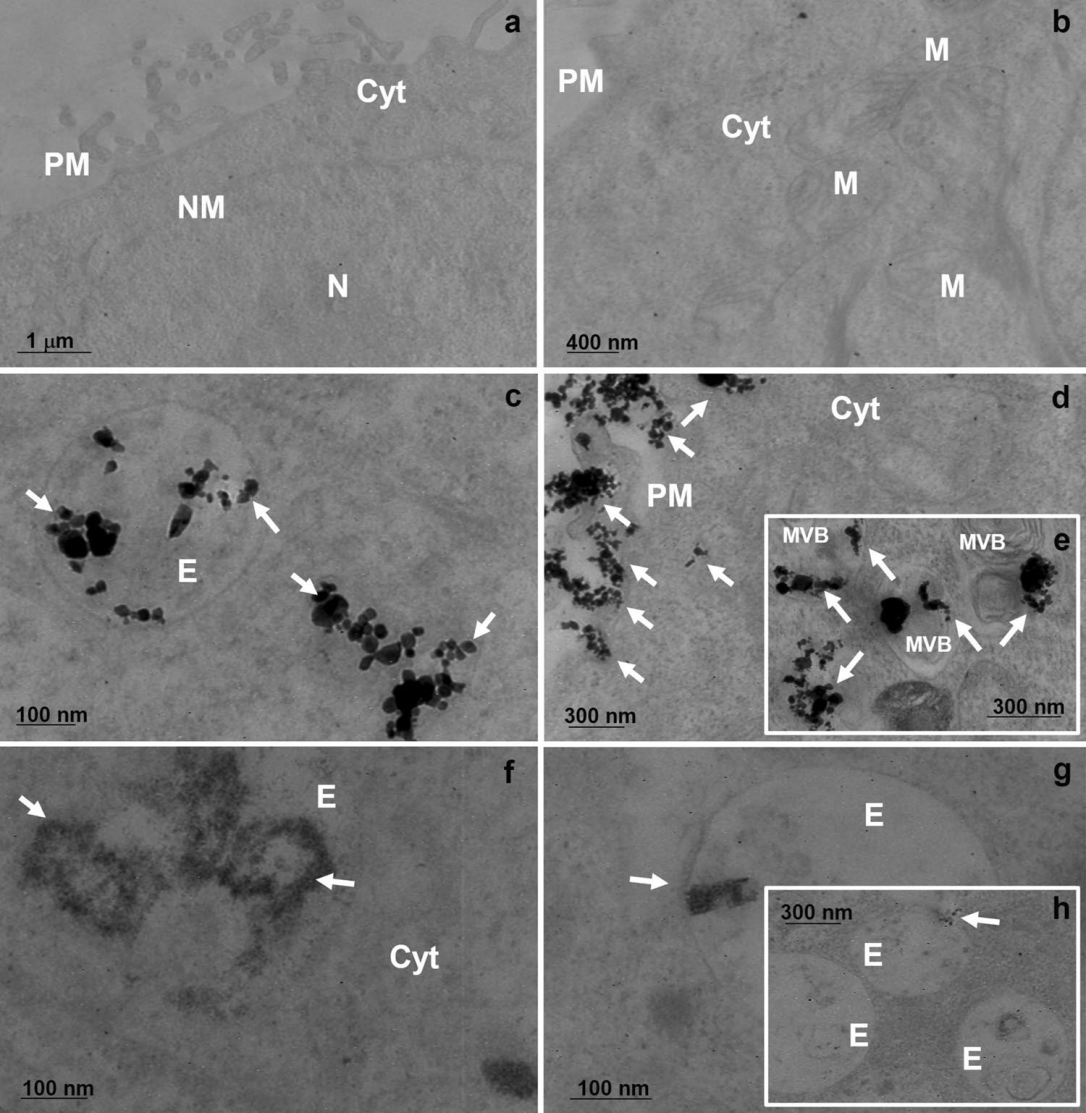
TEM images of A549 exposed to 50 µg/ml CuO NPs. (**a**, **b**) Negative control cells at 1 h and 3 h post-exposure, with structures like plasma membrane (PM), cytoplasm (Cyt), mitochondria (M), nuclear membrane (NM), intact; (**c**) cCuO NPs already accumulated in endosome-like vesicles (E) at 1 h post-exposure; (**d**) cCuO interacting with the PM and entering cells through the endocytic pathway at 3 h post-exposure; (**e**) inset detailing cCuO accumulating in multivesicular bodies (MVBs) at 3 h post-exposure; (**f**) sCuO already internalised in vesicles at 1 h post-exposure and affecting organelle integrity; (**g**) small aggregates of sCuO NPs in endosomes at 3 h post-exposure; (**h**) detail of sCuO interacting with internal membranes. The white arrows indicate the presence of CuO NPs.

**Figure 8:**
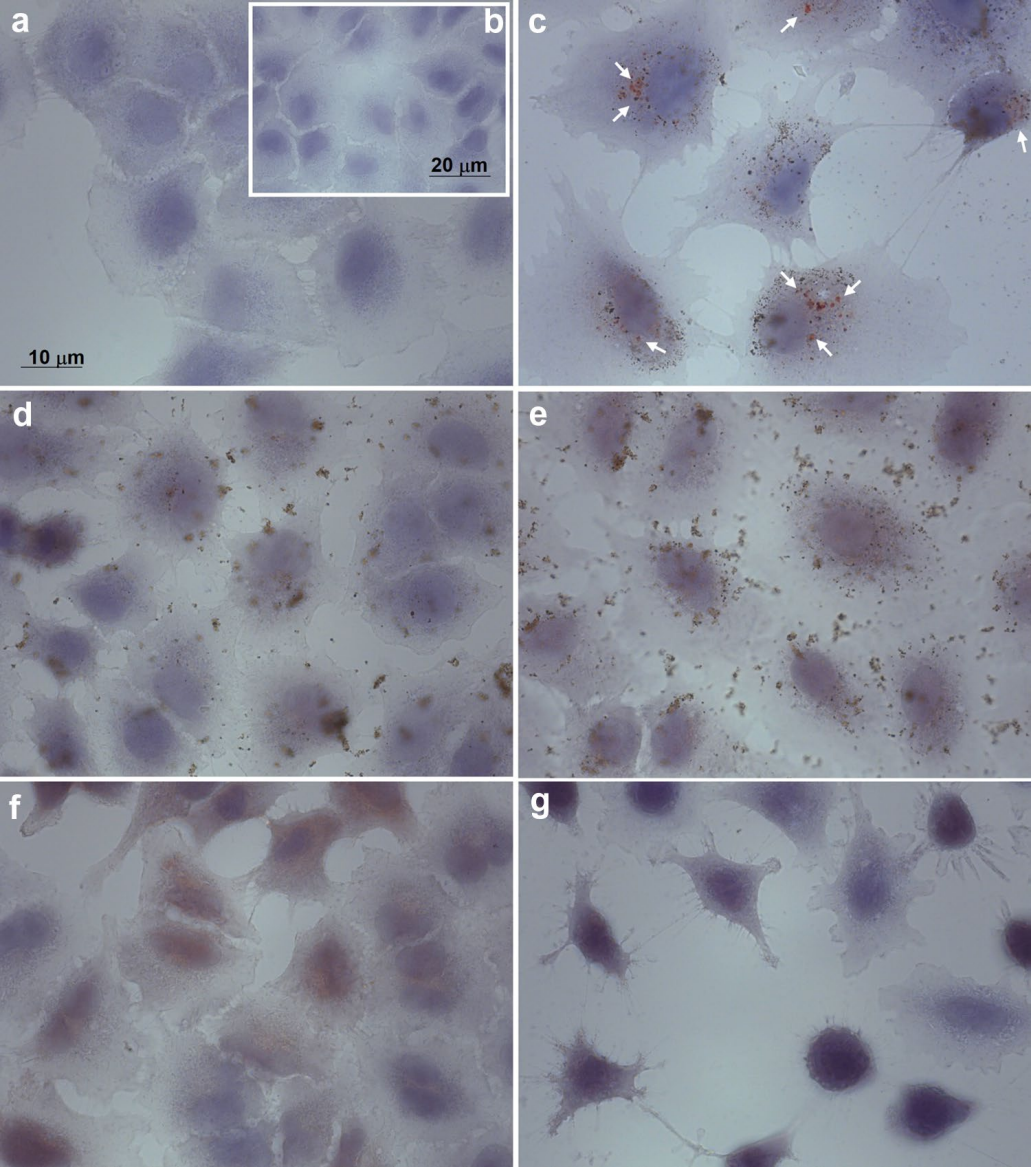
Cytochemistry of intracellular Cu^++^ by Rhodanine staining in A549. (**a**) negative control (unexposed cells); (**b**) Cu^++^ -exposed cells (from CuSO_4_·5H_2_O); (**c**) Positive control (10 µg/ml cCuO-BSA -treated cells, 24 h post-exposure)—red spots (white arrows) testify for the intracellular release of Cu^++^ from NPs; (**d**, **e**) 25 µg/ml cCuO-treated cells at 1 h and 3 h post-exposure, respectively; (**f**, **g**) 25 µg/ml sCuO-treated cells at 1 h and 3 h post-exposure, respectively.

The outer cell morphology and the surface interactions with NPs were analyzed by SEM. When compared to control (Fig. 6a), the cCuO treated cells revealed almost unaltered cell morphology with abundant NP aggregates visible on the outer cell membrane, especially after 3 h of exposure (Fig. 6b, c). TEM imaging demonstrated that, similarly to the negative control (Fig. 7a, b), the ultrastructure of cCuO- exposed cells was preserved as well (Fig. 7c–e), and no NPs engulfed and/or leaking from secondary lysosomes were visible. Comparable to negative- (Fig. 8a) and contrary to positive-control cells (Fig. 8b,c), no lysosomal dissolution of copper after 1 h and 3 h of exposure to cCuO was visualized by the rhodanine staining (Fig. 8d, e; Supplementary Fig. S6).

On the contrary, the cell morphology of A549 cells exposed to sCuO appeared soon affected by the treatment. At 3 h post-exposure very condensed rounded up cells were observed as characteristic morphological effects produced by sCuO (Fig. 6d, e). In addition, very few NPs and NP aggregates were mapped onto cell surface and, when present (Fig. 6f), they were very small sized in comparison with those mapped onto cCuO exposed cells.

The elemental analysis performed by EDX on selected areas, confirmed the particle aggregates as made by Cu for both the particles (see Supplementary Fig. S8).

At high magnification, sCuO NPs internalized in A549 cells already at 1 h post-exposure appeared as aggregates of very small NPs characterized by very irregular and shadowing borders (Fig. 7f). The cell ultrastructure resulted seriously compromised after 3 h exposure (Fig. 7g, h). Under the light microscope, no visible particulate material was appreciable in sCuO NPs treated cells, but a diffuse brownish color was present in the cytoplasm after 1 h exposure (Fig. 8f) and it got even more intense in the condensed apoptotic cells after 3 h exposure (Fig. 8g). However, no clear lysosomal release of copper ions was highlighted by rhodanine staining after exposure to sCuO (Fig. 8f, g; Supplementary Fig S6, S7). This points out that the enhanced biological reactivity of sCuO NPs was associated to their internalization soon after cell exposure, without the involvement of the endo-lysosomal cell digestive pathway. Differently, upon prolonged (24 h) exposure to sublethal concentrations of CuO NPs, the rhodanine staining in A549 cells was characteristically mapped in round-shaped organelles, likely corresponding to secondary lysosomes (Fig. 8c—positive control).

**Figure 9:**
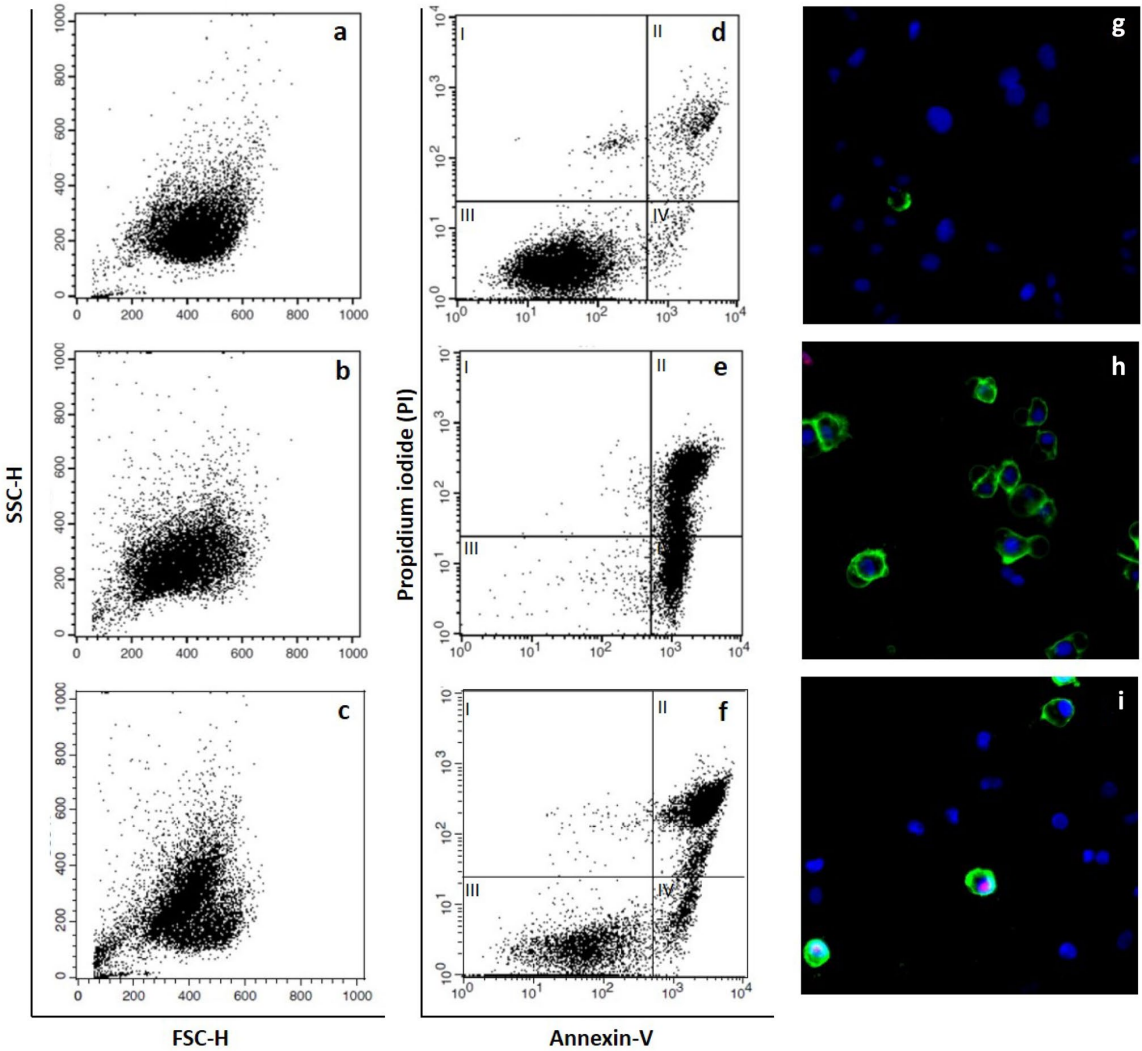
Short-term apoptosis detection (3 h post-exposure) in A549 by flow cytometry and fluorescent microscopy. (**a**–**f**) Representative flow cytometry plots of A549 stained with Annexin V-FITC/PI. (**a**–**c**) forward scattering (FSC-H) versus side scattering (SSC-H); (**d**–**f**) contour diagrams of FITC-Annexin V/PI, log scale; (**g**–**i**) representative pictures acquired by CLSM after A549 Annexin V-FITC/PI/Hoechst staining. (**a**, **d**, **g**) Unexposed cells; (**b**, **e**, **h**) 10 mM H_2_O_2_ -exposed cells; (**c**, **f**, **i**) 50 μg/ml sCuO -exposed cells. The lower left quadrant (III) of each central panel (**d**–**f**) shows the viable cells, which exclude PI and are negative for FITC-Annexin V binding. The upper right quadrants (II) contain dead cells, positive for both FITC-Annexin V binding and for PI uptake. The lower right quadrants (IV) represent the apoptotic cells, FITC-Annexin V positive and PI negative, demonstrating loss of cytoplasmic membrane integrity. Micrographs show viable cells only stained with Hoechst (blue), while apoptotic cells show either double (blu/green) or triple (blu/red/green) staining.

The morphological changes induced by cell exposure to sCuO NPs are characteristic of the early stages of the apoptotic cell death. This was confirmed by both flow cytometry analyses and annexin V-PI staining (Fig. 9). After 3 h of exposure to sCuO NPs, around 70% of the cell population turned out to be positive to annexin V (Fig. 9c,f) compared to untreated cells (Fig. 9, a, d) and in a similar way to H_2_O_2_-exposed cells used as positive control (Fig. 9b,e). On the contrary, no appreciable increase in annexin V-positive cells was registered after exposure to cCuO NPs (data not shown). The flow cytometry measurements of A549 cells exposed to cCuO NPs were sensibly affected by the strong laser side-scattering, likely determined by the abundant cCuO NP aggregates adhering to the cell surface as evidenced already by SEM and TEM analyses (Fig. 6b,c and Fig. 7d).

#### Role of endocytosis and lysosomal activation in cell viability decrease

To identify potential key events in the mechanisms of precocious cell death induced by cCuO and sCuO NPs the roles of particle internalization through endocytosis and lysosomal-enhanced NP dissolution were indirectly investigated by 1) blocking active particle uptake by Cytochalasin D (CytD) and 2) blocking lysosomal acidification by Bafilomycin A1 (BafA1).

CytD is known for its ability of depolymerizing actin filaments thus inhibiting actin-dependent uptake mechanisms like phagocytosis and micropinocytosis^26^. Bafilomycin A1 (BafA1) is considered a strong inhibitor of the lysosomal acidification by blocking the proton pump activity.

**Figure 10:**
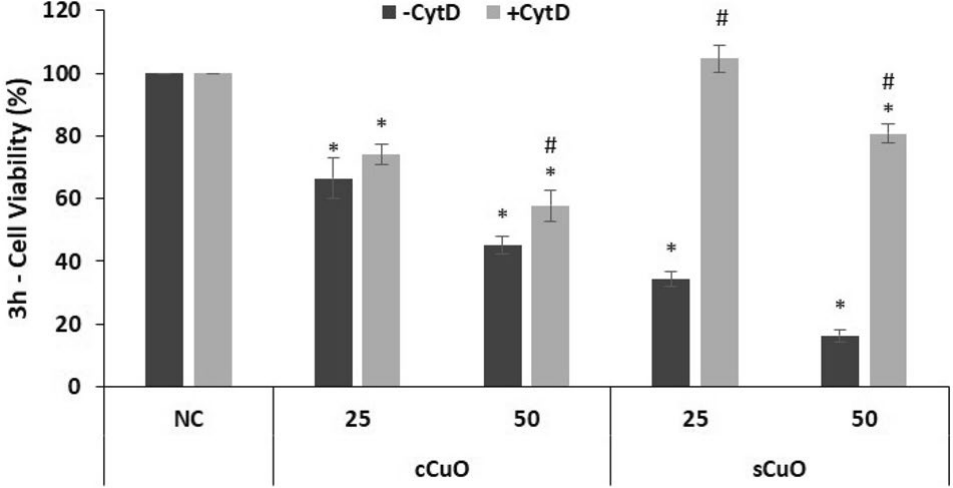
Cell viability results by MTT assay of A549 pre-incubated with an endocytosis inhibitor. Cells were exposed to copper oxide nanoparticles for 3 h after pre-incubation with 4 µM Cytocalasin D (+ CytD, light grey bars) or without pre-incubation (-CytD, dark grey bars). Results are expressed as mean ± SD. *Significantly different from the negative control (NC) (ANOVA + Fisher LSD, p < 0,05). ^#^Significantly different from the correspondent experimental group without inhibitor (t-test, p = 0,002).

**Figure 11:**
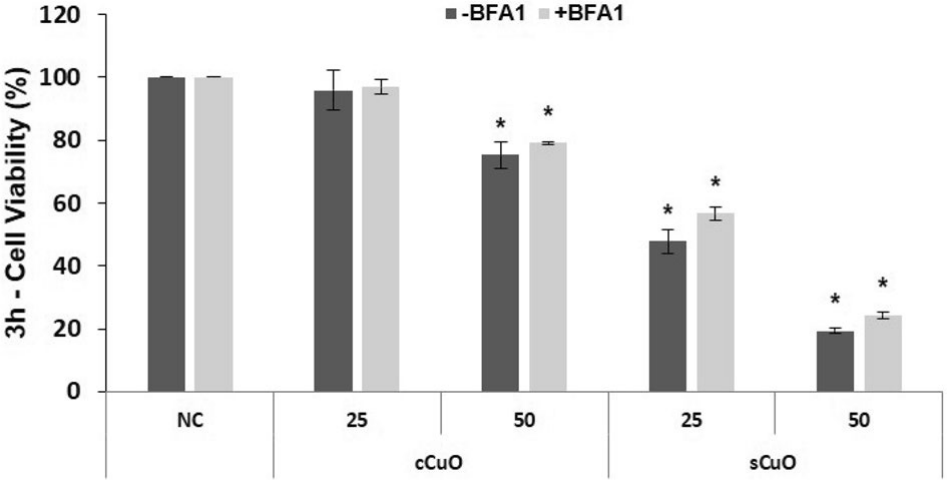
Cell viability results by MTT assay of A549 pre-incubated with a proton-pump inhibitor. Cells were exposed to copper oxide nanoparticles for 3 h after pre-incubation with Bafilomycin A1 (+ BafA1, light grey bars) or without pre-incubation (-BafA1, dark grey bars). Results are expressed as mean ± SD. *Significantly different from the negative control (NC) (ANOVA + Fisher LSD, p < 0,05). #Significantly different from correspondent experimental group without inhibitor (t-test, p = 0,002).

As shown in Figs. 10 and 11, both CytD and BafA1 pre-treatments were not able to rescue cells from death after cCuO exposure, with exception of the slight CytD-induced viability recovery in 50 µg/ml cCuO exposed cells (Fig. 10). On the contrary, a significant toxicity reduction has been observed when cells exposed to sCuO where pre-treated with CytD. However, cells co-incubated with and BafA1 did not show viability recovery in presence of sCuO (Fig. 11).

## Discussion

CuO- and Cu- based nanoparticles have shown exert a strong biological activity (e. g., antibacterial, antifungal, antiviral and anticancer) towards a wide group of pathogenic microorganisms and viruses and this has been already displayed in several studies^27–32^. This characteristic makes CuO commercially applicable in paints, fabrics, agriculture and in production of medical devices used to prevent potential infections^33^. Such wide use and reactivity pose many concerns for human and environmental health and necessary precautions should be taken to reduce the risk related to occupational or environmental exposure.

Different mechanisms of action^34–36^ have been suggested to explain the antimicrobial activity of CuO NPs, which are also responsible for the observed cytotoxicity in mammalian cells.

In the current study, we investigated the precocious cytotoxic mechanisms of two CuO nanoforms, consisting of commercially available and sonochemically synthesized NPs. The aim of the study was to 1) identify specific NP physicochemical features responsible for the toxicity (e. g. crystalline defects, surface reactivity); 2) understand the role of particle internalization and Lysosomal Enhanced Trojan Horse mechanism in inducing precocious cell death; 3) define suitable markers for the NP-related oxidative effects which could be considered as initiating or key events in the view of an AOP-oriented testing strategy.

### Role of physicochemical properties in the precocious effects of CuO NPs

Although both CuO NPs were able to induce a significant and concentration-dependent reduction in cell viability within the first hour of exposure, sCuO resulted to have a greater toxicity compared to cCuO after 3 h and the differences became even more visible after 6 h (see Supplementary Fig. S9). It is well known that the physical and chemical properties of metal-based nanoforms drastically influence their toxic effects, thus the materials used in this study were characterised by combining several techniques.

By TEM, the two CuO NPs appeared to have a significantly different average primary size (cCuO = 33,3 ± 10,7 nm; sCuO = 24,0 ± 3,5 nm). Moreover, they presented a different shape and crystalline structure. cCuO NPs resulted spherical in shape, with a smooth surface and a coherent diffraction volume of tens nanometers. Electron diffraction patterns suggest that their crystallite size is bigger than the one of sCuO, which shows indeed more structural defects. The presence of corners, edges, or defects (increased abrasiveness) were found positively correlated with the increase of particle toxicity, potentially because (i) the increased area helps in adsorption and binding of active compounds and (ii) the increased surface defects also increases the surface area-to-volume ratio which has a direct effect on ROS generation^37^. The high ROS generation potential of sCuO was already highlighted by ESR analysis^2^, confirming that these particles are potentially more hazardous than cCuO when interacting with biological structures^38^.

Also, the primary size, as well as the agglomeration status, may play a role. In a recent paper^12^, CuO NPs with the same primary size as sCuO have been shown inducing higher cytotoxicity in human epithelial cells compared to NPs with the same composition but smaller in size (4 nm), normally considered as more toxic. These findings were partially attributed to the particle size itself, which probably facilitated the internalization and then the intracellular accumulation of NPs.

Besides the size, solubilisation of toxic ions is recognized as a leading mechanism of metal-based NPs effect. After 3 h of incubation in complete culture medium (CCM) in a cell-free experiment, the estimated extracellular ion release from sCuO was around 50% and most of it occurred within the first hour, while only 10% was determined when cCuO (50 µg/ml) was incubated in the same conditions.

Although we found that sCuO is partially soluble in CCM already during the first hours of incubation, the toxicity was practically independent from the extracellular solubility of CuO for both the NPs. Indeed, after exposure to copper ions at the maximum theoretical concentration released by 100 µg/ml CuO NPs (corresponding to 80 µg/ml of soluble copper), we didn’t observe any reduction in cell viability and these findings agree with previous studies in which the cell death induced by Cu^++^ exposure was significantly lower than that produced by NPs^13^.

Considering the dissolution rate, the surface chemistry, and the crystalline properties of sCuO, combined with the precocious effects induced, we can assume that the intrinsic toxic potential related to the sCuO NPs is very high.

### Role of NPs internalization and Lysosomal-Enhanced Trojan Horse mechanism

In the present work, the attention was focused on short-term events and precocious oxidative phenomena that could characterize the cytotoxic mechanism of CuO NPs on A549.

Despite the significant toxicity induced by cCuO and sCuO at 1 h and 3 h post-exposure, the intracellular ion dissolution involving the lysosomal compartment was not found responsible for the observed effects, since we did not detect any lysosomal release of copper ions through the rhodanine staining. Only after an extended exposure period (6 h), sublethal concentrations of cCuO appeared to exploit the Lysosomal-Enhanced Trojan Horse (LETH) mechanism (see Supplementary Fig. S7).

From TEM and DLS analyses, we know that the single particle size and the hydrodynamic diameter of cCuO are bigger than those of the sCuO, suggesting a more rapid deposition onto the cell culture, as also evident by SEM analyses. We may thus hypothesize that, at short-term exposure, endocytosis works for cCuO as temporary and protective mechanism to reduce particle toxicity. Cells try to modulate the stress induced by the interactions with particles through the endocytic process but, in this way, they internalise large amounts of NPs that are going to release ions in the intracellular environment at later stages, which may induce cell death through LETH mechanism (6 h – 24 h)^21^. This interpretation agrees with the results on cell viability obtained by pre-treating the cells with CytD as endocytosis inhibitor^39,40^, where a very slight attenuation of the cCuO-induced cytotoxicity was achieved. Of course, further experiments are required to confirm this hypothesis.

On the contrary, NP interaction/internalization plays a key role to start the cytotoxic process induced by sCuO.

In our study, we observed a significant reduction in cell mortality after co-incubation of sCuO (25, 50 µg/ml) with 4 µM CytD. This evidence confirms that the endocytosis of sCuO particles is playing a pivotal role even in promoting its very early toxicity.

Furthermore, blocking the proton pumps responsible for the lysosomal acidification (by pre-incubating cells with Bafilomycin A1) we did not observe any recovery in term of cell viability after exposure to both CuO NPs at the short exposure times used for this study. Thus, sCuO does not necessarily need to follow the digestive pathway and being dissolved by the extremely acidic lysosomal compartment to exert its toxicity. Indeed, since sCuO NPs have been found quite soluble even in the extracellular environment, they can be even more easily dissolved in other cellular compartments where pH starts to lower, like in early endosomes.

As shown by TEM analyses, the cell ultrastructure looked severely affected by sCuO NPs which, in turn, can be considered responsible of the increased level of oxidized proteins.

Likely, these particles can act very quickly depending on their own surface reactivity (because of size and surface defects) and are more prone to generate free radicals and oxidize macromolecules (DNA, lipids, and proteins), which resulted in a significant oxidative stress and cell death, as also previously observed^41^.

### Precocious oxidative stress and cell death

In a previous study based on A549 cells^15^, CuO NPs have been found localised in both mitochondria and cell nucleus after being taken up through endocytosis. There they stimulated ROS production via impaired electron transport chain, thus inducing structural damage, activation of NADPH-like enzyme system, and depolarization of the mitochondrial membrane^42^, which could definitely result in apoptosis^43,44^.

In our study an increase in intracellular ROS production was detected by fluorescent microscopy in A549 exposed to sCuO (see Supplementary Fig. S10). This was also found being coupled with mitochondrial integrity decrease (see Supplementary Fig. S11). However, ROS alone cannot be considered as reliable marker for precocious oxidative stress due to their transient nature.

As reported in^45,46^, in living cells ROS as H_2_O_2_ and O^2–^ can be converted into the more reactive hydroxyl radical, OH-, which can cause DNA-strand breaks, damage membrane lipids or attack proteins. Proteins are particularly sensitive to the presence of ROS/RNS. The interaction between amino-acids residues with this chemical species or with different intermediates generated by the oxidation of other cellular compounds (as lipids and carbohydrates) could have repercussions on their activity, unfolding, degradation, and, ultimately, on cell functioning^47–51^.

For these reasons, we investigated also CuO NP specific action against thiol groups (thiolation), as suggested in^52^, and carbonylation as potential markers for reversible and irreversible protein modification, respectively.

Here, we have the evidence that CuO NPs are powerful in inducing thiol group oxidation and protein carbonylation in A549 cells, already after short exposure time at high concentration (100 µg/ml) and sCuO retains such effect also at lower concentrations. This testifies once again that sCuO is a stronger oxidating agent compared to cCuO and, additionally, that thiolation could be considered as a good marker for CuO NP precocious biological effects. Our findings agree with^53^ where decline in the levels of NPSH (non-protein thiol groups) and PSH (protein-thiol groups) was also observed in liver and kidney exposed to CuO.

In cells exposed to sCuO we also observed precocious irreversible oxidative damages to biomolecules, as confirmed by the immunochemistry of protein carbonyls since after a 3 h-exposure a significant protein carbonylation increase was detected in A549 cells treated with 50 µg/ml sCuO. Under these exposure conditions, cell size reduction and morphological changes were detected by SEM. Thus, our results suggest that sCuO NPs activate a cell death pathway through an oxidative stress that involves at first proteins and then lipids and is reasonably independent from extracellular and lysosomal release of copper ions, but likely depends on properties and defects of the crystalline structure of the particles, which ultimately dictates the ability to locally generate ROS.

In our study around 70% of the cell population turned out to be positive to annexin V after 3 h exposure to sCuO (by flow cytometry analysis), confirming apoptosis as main mechanism driving to cell death. Usually, cancer cells tend to avoid apoptosis and continue to propagate^45^ but it has been demonstrated that increased ROS level in cancer cells alters the mitochondrial functions and plays a key role in the apoptosis induction^54–56^, opening new opportunities in anticancer therapies. Moreover, Kukia et al.^57^ found apoptosis as main mechanism of cell death in cancer cells exposed to different sized CuO NPs. The involvement of oxidative events in the process driving to cell death in our model was testified by the viability recovery observed in presence of NAC, which works as ROS scavenger.

Co-incubation with N-acetyl cysteine was able to significantly prevent cytotoxicity after exposure to sCuO (25, 50 µg/ml) even if the oxidative insult induced by the highest concentration of NPs was probably too strong to be totally inhibited. Thus, we cannot exclude the possibility of a direct damage provoked by the particles themselves^58^.

Further investigations therefore are needed to understand the potential of sCuO NPs to target specifically cancer cells, preserving the normal ones, in order to evaluate the possibility of applying this material as potential and effective agent in anticancer therapies, in combination with proper surface functionalization. The use of more realistic ad complex models like 3D co-culture systems and organoids, could additionally help in the assessing of this targeting ability.

## Conclusion

Our findings testify that the small size, combined with the high ROS generation potential due to the surface and crystalline characteristics and the capacity of releasing ions independently from the LETH mechanism, makes sonochemically synthesized CuO NPs a powerful cytotoxic agent against A549 cells, when compared to the crystalline and less soluble commercial CuO NPs.

For both the NPs we observed that different mechanisms were competing in inducing cell death, even at very early exposure times. cCuO NPs significantly aggregated and deposited on the top of the cells, potentially inducing mechanical stress. Particle intake through endocytosis, adopted as defence mechanism, besides not rescuing cells from death, contributes to enhance cCuO NP cytotoxicity at later exposure time via the LETH mechanism.

On the contrary, particle intake through endocytosis and the high surface reactivity dictated by the crystalline defects, represented key factors to explain the early cytotoxicity induced by sCuO. Since in addition these NPs have been found quite soluble already in the extracellular environment, they may start to interact and dissolve very quickly also in intracellular environments, like early endosomes. At this level, they may further release copper ions and directly react with the biological structures, thus mining the structural integrity of the vesicles and surrounding cytoplasmic structures. This can be hypothesized as a mechanism precociously affecting cell membranes and the endosomal structure, bringing to the release of the endosomal content, including CuO and Cu^++^, that finally provokes a burst of cell oxidative stress and promotion of cell death pathways.

For applications as cytotoxic or bactericidal agent, the high reactivity of sCuO NPs could represent a great advantage (since with reduced amount of material is possible to obtain high performances), but its surface properties must be seriously considered in the safety-by-design frameworks.

Combined with the minimization of the environmental dispersion and a wiser risk management, the control of the intrinsic biological reactivity of the NPs could be a promising strategy for the implementation of new sCuO embedded or coated materials and devices useful to face the last biological and medical challenges of this century, including the prevention of the pathogens spreading, without disregarding the safety aspects.

## Methods

### NP physical and chemical characterization

Two different nanoforms of CuO have been compared in this work. cCuO (#544,868; CAS Number 1317–38-0), has been purchased from Sigma-Aldrich (Milan, Italy) while sCuO has been kindly provided by Prof. A. Gedanken’s laboratory (Bar-Ilan University, Ramat-Gan, Israel) where it was prepared through sonochemical synthesis.

#### HRTEM analyses

High Resolution Transmission Electron Microscopy (HRTEM) analysis was performed at the Department of Physical Sciences, Earth and Environment of the University of Siena with a Jeol JEM 2010 instrument operating at 200 keV and equipped with slow scan CCD camera Olympus Tengra (2304 × 2304 × 14 bit) for image acquisition. TEM samples were prepared dispersing CuO NPs in isopropylic alcohol, ultrasonicating for 3 min and then dropping 5 µl drop of the solution on Cu-grids supporting a carbon-coated formvar membrane.

#### DLS analyses

CuO NP suspensions were deeply studied by a home-made DLS spectrometer^59^ and the hydrated size of the nanoparticles was evaluated from the autocorrelation functions of the scattered light.

Briefly, NPs in powder form were weighed with a microbalance, suspended in ultrapure water to generate a stock suspension of 8 mg/ml and then sonicated for 5 min in ultrasonication bath working at a frequency of 40 kHz (Sonica – Soltec). Aliquots were pipetted-off the stock suspensions immediately after sonication and diluted in the cell culture medium to obtain working concentrations of 10, 25, 50 and 100 µg/ml. To characterize the hydrodynamic behavior in cell culture medium at 37 °C only the final NP suspensions at 25 and 50 µg/ml have been analysed.

The laser source was a He–Ne laser (HNL210L, Thorlabs, USA) emitting at 633 nm with average power 21 mW. The temperature of a cylindrical quartz cell (Hellma GmbH & Co, Germany) was kept under control by a thermostat (Thermo Haake GmbH, Germany) at 37 °C. The temperature values, monitored by a thermocouple placed just below the scattering cell, had fluctuations smaller than 0.1 °C. The normalized intensity autocorrelation functions (ACFs) were computed by an ISS FCS board (ISS Inc. Urbana, IL, USA) and they were fit to a multi-exponential decay law according to the following relation^60^:

1 $$g^{(2)} (\tau ) = \frac{{\left\langle {I(t + \tau )I(t)} \right\rangle_{t} }}{{\left\langle {I^{2} (t)} \right\rangle_{t} }} = 1 + f\left( {\sum\limits_{k} {A_{k} e^{{ - D_{k} q^{2} \tau }} } } \right)^{2}$$

where *D*_*k*_ is the translational diffusion coefficient of the *k*-th diffusing species, *q* is the wave vector, *q* = (4π n/λ)sin(θ/2), n is the refraction index of the solution, taken equal to that of water at the experimental temperature, λ = 633 nm is the laser light wavelength and θ = 90 deg is the scattering angle. The parameter *f* depends on the ratio between the detector and the coherence area and was left as a free fitting parameter (typically *f* = 0.25–0. 5). The values of *D*_*k*_ provide us the average size of the *k*-th diffusing species, according to the Einstein relation $D_{k} = \frac{{K_{B} T}}{{6\pi \eta R_{k}^{(h)} }}$, where *K*_*B*_ is the Boltzmann constant, *T* and *η* are the solution temperature and viscosity and $R_{k}^{(h)}$ is the hydrodynamic radius of the *k*-th species. We verified that two components with widely different diffusion coefficients were necessary to fit the data. Alternatively, we analyzed the first order correlation functions by applying maximum entropy methods^61^ to obtain the distributions of hydrodynamic radii. In this case, the intensity (second order) correlation function was converted in the first order correlation function (1) and the analysis was performed on *g* ^(1)^ =τ up to the first largest lag-time, τ*, for which *g* ^(2)^ =  τ* < 1. The first order correlation function was fit to a regularized distribution of relaxation rates ($\Gamma = Dq^{2}$) according to^62^:

2 $$g^{(1)} (\tau ) = \int {P\left( \Gamma \right)\exp \left( { - \Gamma \tau } \right)d\ln \left( \Gamma \right)}$$

To determine the surface charge of the particles, NPs were suspended in water and in cell culture medium at the concentrations and with the modalities used for cell exposure. Soon after dispersion, the z-potential was measured using a Malvern Zetasizer.

#### NP extracellular dissolution

To measure the potential copper release during the exposure period, 2 ml (in triplicate) of cCuO and sCuO suspensions prepared in cell culture medium (at the working concentration of 25 and 50 µg/ml), were placed in a 6 well plate and incubated for 1 h and 3 h at 37 °C, 5%, CO_2_ and 95% humidity. Then, each particle suspension was collected and ultra-filtrated by using ultracentrifuge tubes VIVASPIN 20 (Sartorius Stedim Biotech GmbH, Goettingen, Germany) with a molecular weight cut-off of 10,000 Da. The supernatants were carefully separated by the pellet, acidified with HNO_3_ (2% final) and analyzed through ICP-OES Optima 7000 DV (Perkin-Elmer, Santa Clara, CA, USA) to measure the Cu^++^ released in the medium.

### Cell cultures and treatments

The in vitro studies were performed using the human alveolar epithelial cell line A549 (American Type Culture Collection, ATCC).

A549 cells were grown and maintained until exposure in OptiMEM 1X medium supplemented with heat-inactivated 10% foetal bovine serum and 1% mixture of antibiotics (Penicillin Streptomycin 5000 µg/ml), at 37 °C, in a 5% CO_2_ humidified atmosphere. All The reagents for cell culture were purchased from Gibco (Thermo-Fischer).

The exposure has been performed in submerged conditions on cells at 70–80% confluence, by administrating the CuO NPs suspensions prepared in sterile MilliQ water and then diluted in cell culture medium in order to generate the final NP working concentrations of 10, 25, 50 and 100 µg/ml. The stock NP suspensions have been freshly prepared and vortexed 30 s just before dilution in cell culture medium. The time points selected to study the precocious effects induced by the two compounds have been 1 h and 3 h.

To reduce as much as possible the interference of exogen protein adsorption onto NP surface, BSA- and serum-free cell culture medium was used to prepare the NP working suspensions.

### Cell viability

A549 cells were seeded at a density of 1,6 × 10^5^/well in 6 multi-well plates for 24 h. Then they were exposed to increasing concentrations of cCuO and sCuO (10 ÷ 100 µg/ml) for 1 h and 3 h. At the end of the exposure time the cell viability was assessed by MTT assay. In parallel, similar experiments were performed by pre-incubating the cells with different antioxidants/inhibitors to verify whether the presence of this substances could have a beneficial effect on the cell survival.

Specifically, 10 mM N-acetyl-L-cysteine (Sigma-Aldrich; Milan, Italy) was used as antioxidant and added to the cell culture medium 30 min before the exposure to CuO NPs and kept along the duration of the treatment.

4 µM Cytocalasin D (Sigma-Aldrich; Milan, Italy) was used as endocytosis inhibitor and added to the exposure medium 1 h before the treatment with CuO NPs.

Pre-treatment for 1 h with 50 nM Bafilomycin A1 (Sigma-Aldrich; Milan, Italy) was applied to inhibit apoptosis.

Negative control cells were incubated in cell culture medium spiked with sterile MilliQ water to reproduce the same volume of stock suspensions presents in the medium of the CuO NP-exposed cells. In addition, the contribution to cytotoxicity of copper ions potentially released from NPs in the cell culture medium was also evaluated. To do that, cells were exposed to a CuSO_4_∙5H_2_O solution prepared in cell culture medium at a nominal Cu^++^ concentration corresponding to the maximum theoretical release from the highest NP concentration tested (100 µg/ml).

At the end of each exposure, the culture medium was replaced with fresh medium containing MTT solution (0.3 mg/ml) and cells were incubated for additional 2.5 h at 37 °C, 5% CO_2_. The formazan crystals formed were dissolved in dimethyl sulfoxide (DMSO). The plates were shacked for 10 min at room temperature and the optical density was measured by a multiplate reader (Multiskan Ascent, Thermo Electron Corporation, Vantaa, Finland) at 570 nm, using 690 nm as a reference wavelength. The results were representative of at least three independent experiments by using three technical replicates each time.

### Cell oxidative markers

Anti-dinitrophenyl-KLH (anti-DNP) antibodies, rabbit IgG fraction, goat anti-rabbit IgG, and goat anti-mouse antibodies conjugated with Alexa 488 were purchased from Molecular Probes (Eugene, OR, USA). ECL Plus Western blotting detection reagents were obtained from GE Healthcare (Milan, Italy). Precision Plus Protein All Blue Standards, ranging from 10 to 250 kDa, were obtained from Bio-Rad Laboratories s.r.l. (Segrate, Italy). Biotin-maleimide (N-biotinoyl-N′-(6-maleimidohexanoyl) hydrazide), purchased from Sigma-Aldrich (Milan, Italy) as well as all the other reagents of analytical grade.

A549 cells were seeded at a density of 1,6 × 10^5^/well in 6 multi-well plates for 24 h and exposed for 1 h and 3 h to CuO NPs (or Cu^++^ solution) under the same conditions applied to determine the metabolic activity by MTT assay. 10% cigarette smoke extract (CSE) prepared as previously described^63^, and 500 µM HOCl were used as positive controls for protein carbonylation and protein SH-group oxidation, respectively.

For both protein carbonylation sulphydryl oxidation at the end of the exposure cell monolayers were rinsed two times in PBS, recovered by mechanical scraping and lysed in RIPA buffer added with a protease inhibitors cocktail. Cell lysates were centrifuged at 11,000 rpm for 4 min and then sonicated 3 times for 10 s each on ice-bath, using a Sanyo Soniprep ultrasonicator (5 µM amplitude).

Samples were centrifuged at 20,000 g for 5 min to remove potential NPs left, and the supernatants were collected and immediately stored at -20 °C.

#### Protein carbonylation

Carbonylated proteins were derivatized with 2,4-dinitrophenylhydrazine (DNPH). 200 µg proteins in lysis buffer (final concentration 1 mg/ml) were mixed with 40 µl of 10 mM DNPH in 2 N HCl and incubated for 60 min in the dark with frequent vortexing. After derivatization, protein samples were mixed with 240 µL of 20% trichloroacetic acid (TCA) and incubated for 10 min in ice. After centrifugation at 20,000 g per 15 min at 4 °C, protein pellets were washed three times with 1:1 ethanol/ethylacetate to remove free DNPH. After air-drying, pellets were resuspended in 2 × reducing Laemmli sample buffer. Proteins were separated by SDS-PAGE (12% Tris–HCl resolving gel) and transferred to polyvinylidene difluoride (PVDF) membrane. Derivatized proteins were detected by Western immunoblotting with anti-dinitrophenyl-KLH (anti-DNP) antibody. Specifically, PVDF membrane was washed in PBST (10 mM Na phosphate, pH 7.2, 0.9% (w/v) NaCl, 0.1% (v/v) Tween-20) and blocked for 1 h in 5% (w/v) non-fat dry milk in PBST. After washing three times with PBST for 5 min each, carbonyl formation was probed by a 2 h- incubation with 5% milk/PBST containing anti-DNP antibodies (1:40,000 dilution). After three washes with PBST for 5 min each, the membrane was incubated with a 1:80,000 dilution of the secondary antibody linked to horseradish peroxidase (HRP) in 5% milk/PBST for 1 h. After washing three times with PBST for 5 min each, immunostained protein bands were visualized with enhanced chemiluminescence detection (ECL).

#### Immunocytochemical detection of protein carbonyls

A549 cells were seeded on sterilized glass coverslips placed in 6-well plates, at the same density used for the previous experiments. After 3 h of exposure to 25 and 50 µg/ml of cCuO and sCuO, cell monolayers were fixed in methacarn, derivatized with DNPH solution (0.1% v/v in 2 M HCl for 1 h) and unspecific sites were blocked with NGS (Molecular Probes). Cells were then incubated with the monoclonal anti-dinitrophenyl antibody (Sigma, Saint Louis, USA) and the signal retrieving was achieved by an AlexaFluor-488 conjugated goat anti-mouse secondary antibody (Molecular Probes). Nuclei were stained with 5 µM DRAQ5™ (Thermo-Fisher Scientific).

Slides were finally mounted in Prolong Gold Anti-fade mounting medium (Molecular Probes) and observed with a Leica TCS SP5 confocal microscope. Reflected-light optics were used at a magnification of 40x (1.25 NA Plan-Apochromat). Samples were illuminated with a 488 nm Argon/Krypton laser, using an intensity of the AOTF filter by 10%. Images were processed with the Leica dedicated LAS AF software.

#### Protein Sulphydryl Oxidation

Sample proteins were derivatized with biotin-maleimide. 100 µg proteins in lysis buffer (final concentration 1 mg/ml) were mixed with 75 µM of biotin-maleimide and incubated for 60 min in the dark with frequent vortexing. After derivatization, protein samples were mixed with a volume of 2 × reducing Laemmli sample buffer. Proteins were separated by SDS-PAGE (12% Tris–HCl resolving gel) and transferred to PVDF membrane. Derivatized proteins were detected by Western blotting. PVDF membrane was washed in PBST and blocked for 1 h in 5% (w/v) non-fat dry milk in PBST. After washing three times with PBST for 5 min each, protein sulphydryl oxidation was probed by a 2 h- incubation with 5% milk/PBST containing streptavidin linked to HRP (1:5,000 dilution). After three washes with PBST for 5 min each stained protein bands were visualized with ECL. Biotinylated and carbonylated proteins were visualized by enhanced chemiluminescence (ECL) detection (Bio-Rad, cod. 1,705,061) using the ChemiDoc Touch Imaging System (Bio-Rad). ECL signals were normalized on PVDF Amido Black staining. Densitometric analysis of both protein carbonylation and sulphydryl oxidation was performed by scanning the chemiluminescence film images and using the Image J 1.40d software (National Institutes of Health, USA).

### Copper cytochemistry

Intracellular copper ion dissolution was determined by a cytochemical method using rhodanine. A549 cells were seeded on sterilized glass coverslips placed in 6-well plates, at the same density used for the previous experiments. After exposure for 1 h and 3 h to 25 and 50 µg/ml of cCuO and sCuO, they were fixed in buffered formalin (4%) and incubated with 0.12 g/l rhodamine (p-Dimethylaminobenzylinene-rhodanine) alcoholic solution. Then they were abundantly rinsed with MilliQ water. Nuclei were counterstained with haematoxylin. Slides were mounted with a glycerol-based medium and immediately observed under the light microscope (Axioplan—Zeiss).

### Cytofluorimetric analysis of cell death

This analysis was performed both by flow cytometer and fluorescence microscope according to the instructions on the manufacturer. For flow cytometer analysis cell were seeded directly on 6 well plates at the density of 1,6 × 10^5^/well. Briefly, after 3 h of exposure to both CuO NPs, cells were washed twice with PBS, detached using trypsin and then incubated with the Annexin V-Alexa Fluor® 488 Conjugate (Thermo-Fisher Scientific) and/or propidium iodide (PI) reagent for 15 min in the dark. Positive controls were treated with 10 mM H_2_O_2_. The fluorescence signal was analysed by a Becton Dickinson FACS Calibur flow cytometer immediately after the adding of 400 μgl of 1X Annexin binding buffer to each sample.

For the microscopic evaluation, cells were grown on sterile glass coverslips placed in 6 well plates, then exposed to NPs and incubated with the Annexin V-Alexa Fluor® 488 Conjugate and/or PI reagents. Coverslips were mounted on glass slides and immediately observed under a Zeiss Axio-Observer Z1 equipped with an AxioCam MRc. Digital images were elaborated by the dedicated AxioVision Rel. 4.8 software.

### Electron microscopy

SEM analyses were performed mainly to investigate cell-particle interactions on the cell surface. Cell monolayers grown on sterile glass coverslips were fixed in 2% glutaraldehyde prepared in cacodilate buffer, post-fixed with 1% OsO_4_ (Sigma-Aldrich; Milan, Italy) first dehydrated in a graded ethanol series followed by a Hexamethyldisilazane (HMDS) graded series (25–50–75–100%). Coverslips were then mounted onto standard SEM stubs, coated with pure gold and observed under a Zeiss LEO 1430 scanning electron microscope operating at an accelerating voltage of 20 kV.

TEM analyses were conducted to study the ultrastructural modifications and to visualize the NPs in the subcellular compartments. A549 were seeded at a density of 1,35 × 10^5^ in ø3.5 cm sterile plastic petri dishes and 24 h later exposed to CuO NPs. At the end of the exposure time the monolayers were fixed in 2.5% glutaraldehyde in cacodilate buffer, post-fixed with 1% OsO_4_ and routinely embedded in EPON-Araldite resin. Frontal 60 nm ultrathin sections of the cell layers were cut on a Reichert Jung ultramicrotome, collected onto 300 mesh copper grids, and finally observed with a Jeol JEM1220 transmission electron microscope, operating at an accelerating voltage of 80 kV and equipped with a Lheritier digital camera. To prevent metal salt contamination (potentially generating artifacts) and allow a clear NP visualization in cells, pre- and post-embedding stains were avoided.

### Statistics

All data are expressed as mean ± standard deviation (SD) of at least three experimental replicates by using three technical replicates each time, unless otherwise specified. Statistical analysis was done using SigmaStat and GrapPad Prism9 software. A one-way analysis of variance (ANOVA) with a subsequent Fischer’s LSD post-hoc test was performed for multiple comparisons. Differences between mean values (compared to the negative controls) were considered statistically significant when p < 0,05 (*). Differences among groups were considered statistically significant when p < 0,05 by t-test (#).

## Supplementary Information

Supplementary Information.

## Data Availability

Authors can confirm that all relevant data are included in the article and/or its Supplementary Information file. Detailed datasets used and/or analysed during the current study are available from the corresponding author [PM] on reasonable request.
