# Supplementary material for: Biological mechanism of cell oxidative stress and death during short-term exposure to nano CuO
Source: Sci Rep. 2023 Feb 9;13:2326. doi: 10.1038/s41598-023-28958-6 (PMC9911756; doi:10.1038/s41598-023-28958-6)
Supplement: Supplementary file 1 — Supplementary Information. [file 41598_2023_28958_MOESM1_ESM.docx]

**SUPPLEMENTARY INFORMATION**

**Biological mechanism of cell oxidative stress and death during short-term exposure to nano CuO**

Elisa Moschini^1,4^, Graziano Colombo^2^, Giuseppe Chirico^3^, Giancarlo Capitani^1^, Isabella Dalle-Donne^2^, Paride Mantecca^1*^

*^1^ Department of Earth and Environmental Sciences, Research Center POLARIS, University of Milano Bicocca, 1 Piazza della Scienza - 20126 Milan, Italy*

*^2^ Department of Biosciences, Università degli Studi di Milano, 26 via Celoria - 20133 Milan, Italy*

*^3^ Department of Physic, University of Milano Bicocca, 2 Piazza della Scienza - 20126 Milan, Italy*

*^4^ Luxembourg Institute of Science and Technology (LIST), Environmental Research and Innovation (ERIN) Department, 41, rue du Brill, L-4422 Belvaux, Grand-Duchy of Luxembourg*

**Particle size and particle size distribution by transmission electron microscopy**

Particle size was determined by TEM imaging on 5µl droplets of an appropriate dilution made from the NP stock dispersions prepared in water. Each droplet was placed onto Formvar©-coated 200 mesh copper grids, allowed to deposit for 3 min and dried by blotting the water in excess with filter paper. Once dried, grids were inserted in a Jeol JEM-1220 electron microscope and observed at an acceleration voltage of 100KV. Digital images were taken with a Gatan CCD camera. Around 300 NPs were measured by using the specific software. A statistical approach was applied to remove potential outliers (ROUT test, Q=0.2%; GraphPad Prism9).

The measured average size for cCuO and sCuO was 33.3±10.7 nm and 24.0±3.5 nm, respectively (the size is expressed as mean±SD).

Supplementary Figure S1 and S2 show the size frequency distribution for cCuO NPs and sCuO NPs respectively. The population of cCuO particles was quite heterogeneous (10-100nm) but only very few NPs were found to be close to 100 nm, while sCuO NPs distribution was very narrow as shown also in Supplementary Figure S3. The statistical comparison of the mean values for the two compounds revealed that the means are statistically significantly different (t-test with Welch's correction, p<0,0001).


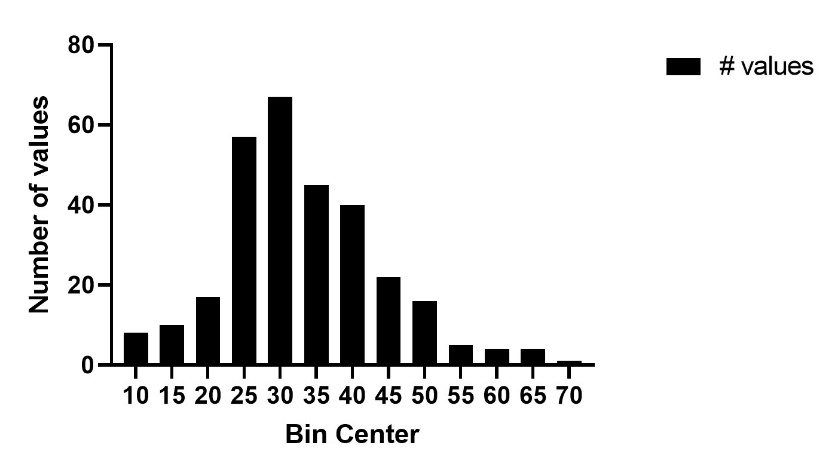


Supplementary Figure S1. Size frequency distribution of cCuO nanoparticles after removal of outliers through the ROUT test (Q=0.2%). Data is represented as number of values found in each category (bin width = 5nm).


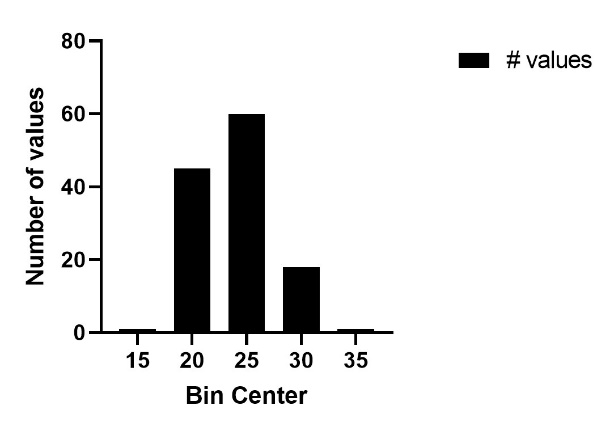


Supplementary Figure S2. Size frequency distribution of sCuO nanoparticles. Data is represented as number of values found in each category (bin width = 5nm).


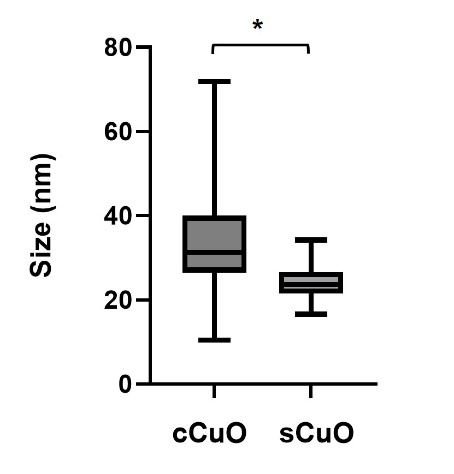


Supplementary Figure S3. Box plot showing size distribution comparison between cCuO and sCuO. *Statistically significantly different (t-test with Welch's correction, p<0,0001).

**Dynamic Light Scattering characterization of the nanoparticles**

Analysis of the second order correlation function of the light scattered by suspensions of the cCuO and sCuO nanoparticles

The NP suspensions were studied on a home-made DLS spectrometer [60]. The laser source was a He-Ne laser (HNL210L, Thorlabs, USA) emitting at 633 nm with average power 21 mW. The scattering angle was set at 90°. A cylindrical quartz cell (Hellma GmbH & Co, Germany) was thermostated (Thermo Haake GmbH, Germany) at 37 °C. The temperature monitored by a thermocouple placed just below the scattering cell had fluctuations smaller than 0.1 °C. The normalized intensity (second order) autocorrelation functions (ACFs) were computed by an ISS FCS

board (ISS Inc. Urbana, IL, USA) and they were fit to a multi-exponential decay law according to the following relation [61]:

$g^{(2)}(\tau)=\frac{\left\langle I(t+\tau)I(t)\right\rangle_{t}}{\left\langle I^{2}(t)\right\rangle_{t}}=1+f\left(\sum_{k} A_{k}e^{-D_{k}q^{2}\tau}\right)^{2}$ (1)

Supplementary Table 1. Best fit parameters of the double Exponential analysis of the second order ACFs.

| **Sample** | **Radius first component (nm)** | **Radius first component (nm)** | **Relative amplitude first component** |
| --- | --- | --- | --- |
| **sCuO 25 µg/ml** | 210 ± 2 | 6300 ± 600 | 88% |
| **sCuO 50 µg/ml** | 190 ± 2 | 790 ± 200 | 77% |
| **cCuO 25 µg/ml** | 270 ± 10 | 4500 ± 1200 | 77% |
| **cCuO 50 µg/ml** | 770 ± 70 |  | 100% |

The hydrodynamic radii were computed according to the Stokes-Einstein relation $D_{k}=\frac{K_{B}T}{6\pi\eta R_{k}^{(h)}}$, where *K_B_* is the Boltzmann constant, *T* and η are the solution temperature and viscosity and $R_{k}^{(h)}$ is the hydrodynamic radius of the *k*-th species.

Supplementary Figure S4. The double exponential fit of the second order (intensity) ACFs for the sCuO (panel A) and the cCuO (panel B) NPs. For each concentration (either 25 or 50 μg/ml two distinct ACF acquisition (open symbols) with the corresponding best fit functions (colored solid lines) are plotted.

Maximum Entropy analysis of the first order ACFs

As a second approach to the analysis of the ACFs from the nanoparticle suspensions, the first order ACFs $g^{(1)}\left( \tau\right)$ were computed from the measured second order ACF, $g^{(2)}\left( \tau\right),$ as $g^{(1)}\left( \tau\right)= \sqrt{g^{(2)}\left( \tau\right)-1}$, and the analysis was performed on $g^{(1)}\left( \tau\right)$ up to the first largest lag-time, $\tau^{*}$, for which $g^{(2)}\left( \tau^{*} \right)$< 1. The first order correlation function was fit to a regularized distribution of relaxation rates ($\Gamma=Dq^{2}$) according to [62]:

$g^{(1)}(\tau)=\int P\left(\Gamma\right)\exp\left(-\Gamma\tau\right)d\ln\left(\Gamma\right)$ (2)

In this second approach, a single component was always found in the distributions of relaxation rates $\Gamma$. However, the distributions always displayed a marked skewness at high radii value that is accounted for by additional minor components (up to 4 components in total). The distributions $P\left( \Gamma\right)$ were then fit to a composition of up to 4 log-normal functions $P\left(\Gamma\right)=\sum_{k=1,.4} A_{k}\exp\left(-\frac{\ln^{2}\left(\Gamma/\Gamma_{k}\right)}{2\sigma_{k}^{2}}\right)$.

We have then obtained a weighted average of the hydrodynamic radius according to the relation:

$\frac{1}{\left\langle R\right\rangle}=\sum_{k} \frac{A_{k}}{R_{k}}$ (3)

Supplementary Table 2. Best fit average hydrodynamic radii of the maximum entropy analysis of the first order ACFs for the copper oxide NPs.

| **Sample** | **Average Radius (nm)** |
| --- | --- |
| **sCuO25** **µg/ml** | $200_{130}^{300}$ |
| **sCuO50 µg/ml** | $420_{270}^{600}$ |
| **cCuO25 µg/ml** | $380_{270}^{600}$ |
| **cCuO50 µg/ml** | $700_{570}^{900}$ |

The uncertainties in the radii were computed according to the relation:

$\frac{1}{\sigma^{2}}= \sum_{i} \frac{1}{\sigma_{i}^{2}}$ (4)

The super and subscripts in Supplementary Table 2 correspond to the minimum and maximum variability of the radius according to the average log-normal distribution.

Supplementary Figure S5. Exemplary distributions of radii for the suspension of Nanoparticles. T=37 °C. The solid line is a multicomponent lognormal fit to the data. The ACFs for the 25 and 50 μg/ml suspensions are reported in filled and open symbols, respectively. Panel A: sCuO NPs. Panel B: cCuO NPs.

**Copper cytochemistry**

Intracellular copper ion dissolution was determined by a cytochemical method using rhodanine. High concentration of CuO NPs (50 µg/ml) was applied to A549 for short exposure time (1 h and 3 h) while lower concentrations (10 µg/ml and 25 µg/ml) were kept in contact with cells for 6 h. After exposure cells were rinsed, formalin fixed, and incubated with 0.12 g/l rhodanine (p-Dimethylaminobenzylinene-rhodanine) alcoholic solution. Abundantly rinsing was done and nuclei were counterstained with haematoxylin. Slides were mounted in a glycerol-based medium and immediately observed under the light microscope (Axioplan - Zeiss).

Results from this test are summarised in Supplementary Fig. S6 and S7.


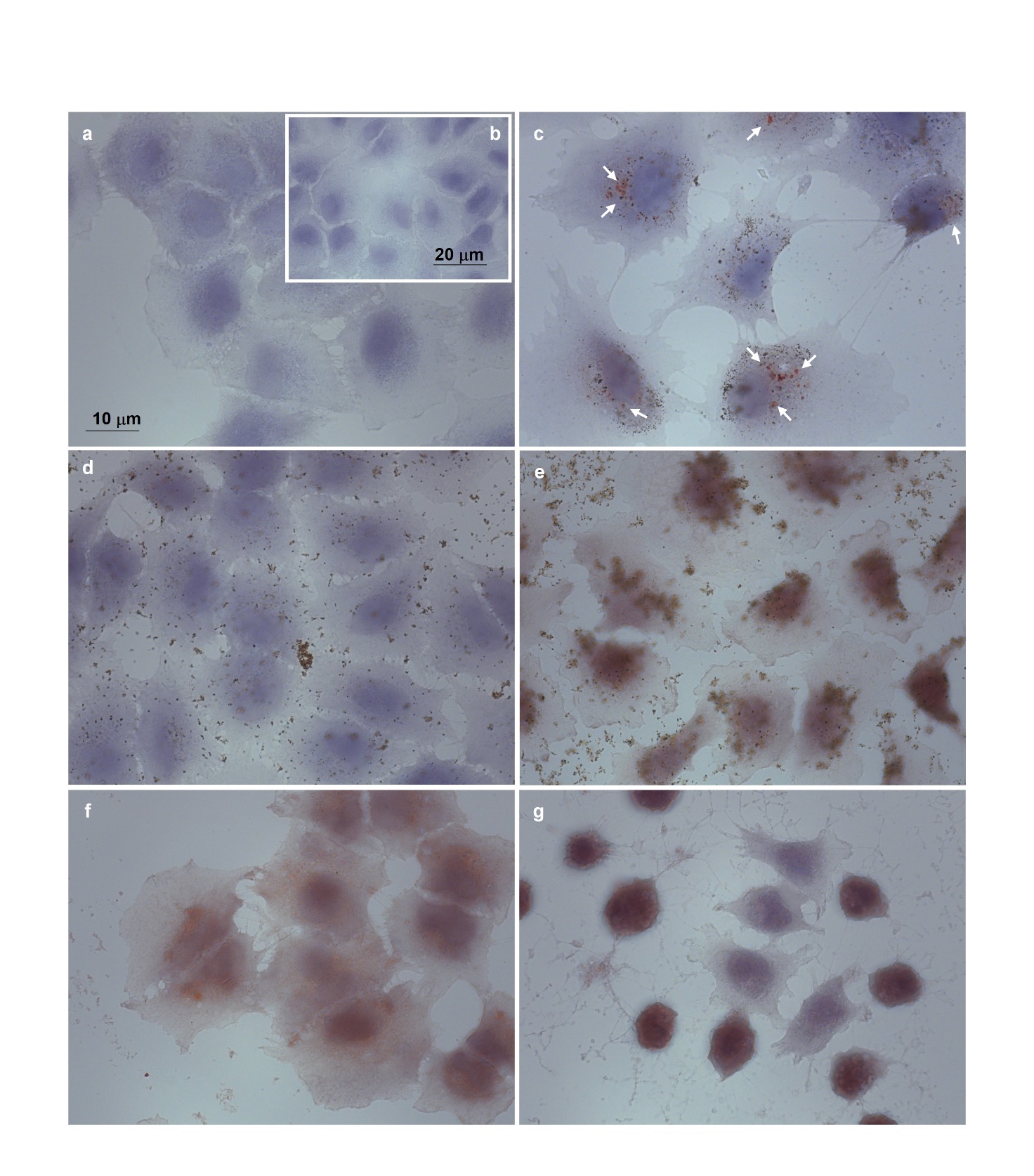
Supplementary Figure S6. Cytochemistry of Cu^++^ by Rhodanine staining in A549. a) negative control (unexposed cells); b) Cu^++^ -exposed cells (from CuSO_4_·5H_2_O); c) Positive control (10 μg/ml cCuO-BSA -treated cells, 24 h post-exposure) - red spots (white arrows) testify for the intracellular release of Cu^++^ from NPs; d, e) 50 μg/ml cCuO-treated cells at 1 h and 3 h post-exposure, respectively; f, g) 50 μg/ml sCuO-treated cells at 1 h and 3 h post-exposure, respectively.


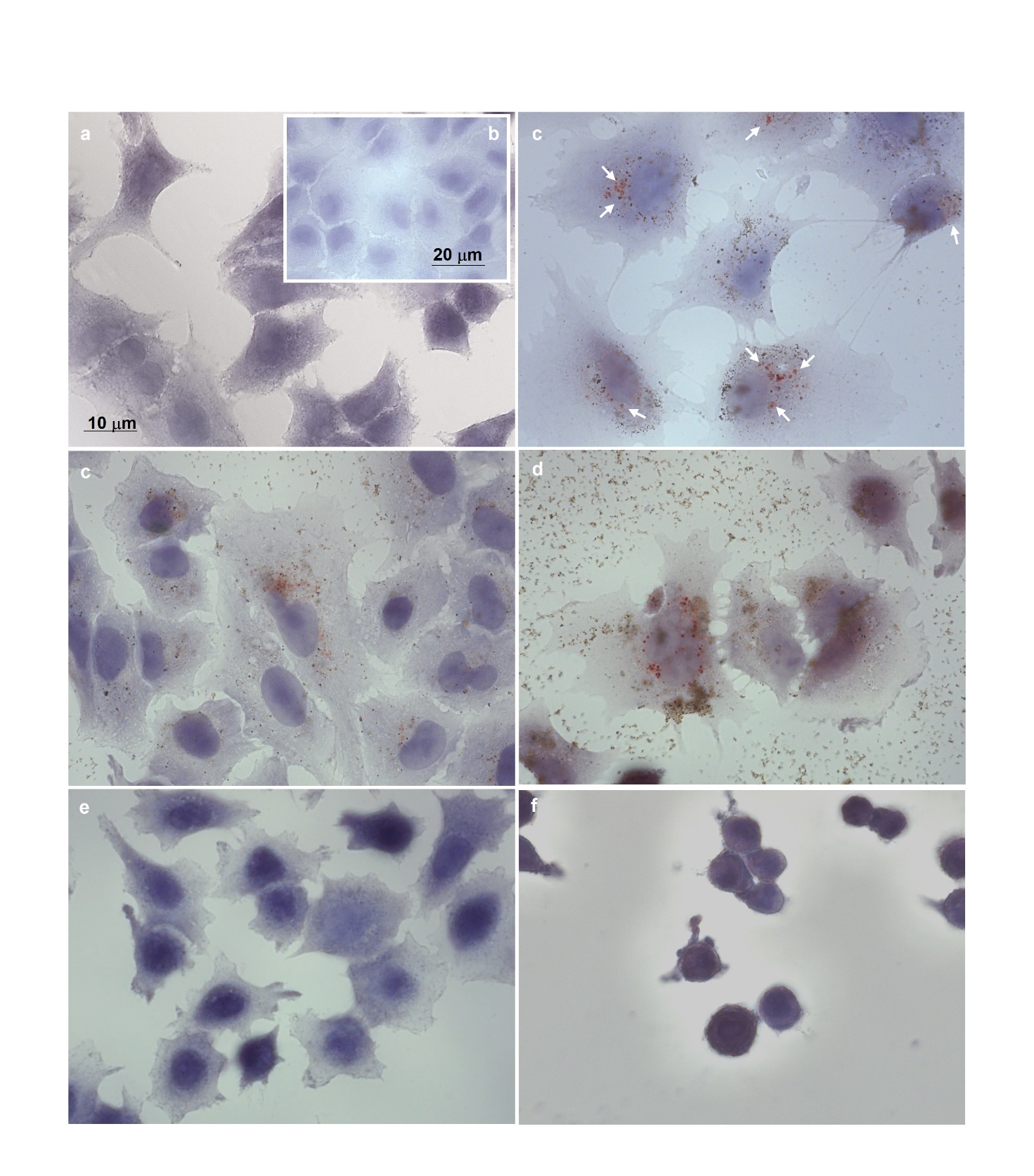


Supplementary Figure S7. Cytochemistry of Cu^++^ by Rhodanine staining in A549. a) negative control (unexposed cells); b) Cu^++^ -exposed cells (from CuSO_4_·5H_2_O); c) Positive control (10 μg/ml cCuO-BSA -treated cells, 24 h post-exposure) - red spots (white arrows) testify for the intracellular release of Cu^++^ from NPs; d, e) 25 and 50 μg/ml cCuO-treated cells at 6 h post-exposure, respectively; f, g) 25 and 50 μg/ml sCuO-treated cells at 6 h post-exposure, respectively.

**EDX analysis**

Cell monolayers grown on sterile glass coverslips and exposed to cCuO and sCuO NPs were fixed in 2% glutaraldehyde prepared in cacodilate buffer, post-fixed with 1% OsO_4_ (Sigma-Aldrich; Milan, Italy) first dehydrated in a graded ethanol series followed by a Hexamethyldisilazane (HMDS) graded series (25–50–75–100%). Coverslips were then mounted onto standard SEM stubs, coated with pure gold and observed under a Zeiss LEO 1430 scanning electron microscope operating at an accelerating voltage of 20 kV. EDX spectra were acquired on specifically selected areas of samples to characterise their elemental composition. Supplementary Fig. S8a shows the spectrum acquired from cCuO exposed cells (Fig. 6c of the main manuscript) while Supplementary Fig.S8b shows the spectrum of a detail acquired on sCuO exposed cells (Fig 6f of the main manuscript). On both the spectra is visible the characteristic peak of copper (Cu) confirming the presence of CuO nanoparticles on cell surface.

*
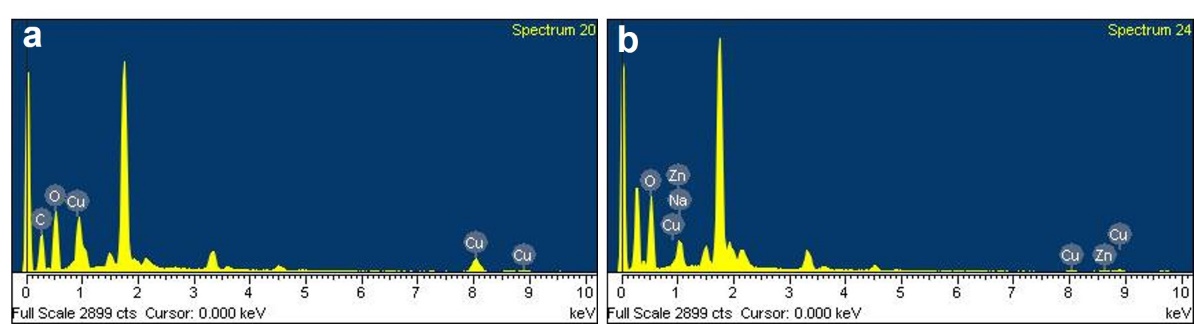
*

Supplementary Figure S8. EDX spectra acquired on A549 exposed for 3 h to cCuO (a) and sCuO (b). The spectra refer respectively to the samples shown in Fig 6 c, f in the main manuscript.

**Cell viability**

The viability was assessed by MTT assay in cells exposed to cCuO and sCuO at 10÷100 µg/ml for 6 h. At the end of exposure, culture medium was replaced with new medium containing MTT solution (0.3 mg/ml) and incubated for 2.5 h at 37°C. Formazan crystals formed were dissolved in dimethyl sulfoxide (DMSO). The plates were shacked for 10 minutes at room temperature and optical density was recorded at 570 nm (using 690 nm as a reference) using a multiplate reader (Multiskan Ascent, Thermo Electron Corporation, Vantaa, Finland). Untreated cells were used as negative control.

The data, shown in Supplementary Figure S9, are representative of at least three independent experiments. Statistical comparisons were performed by one-way ANOVA followed by Fischer’s LSD test.

The contribution to cytotoxicity of copper ions dissolved from NPs was also evaluated at the maximum theoretical dissolving concentration for the higher NP concentration tested (100 µg/ml).


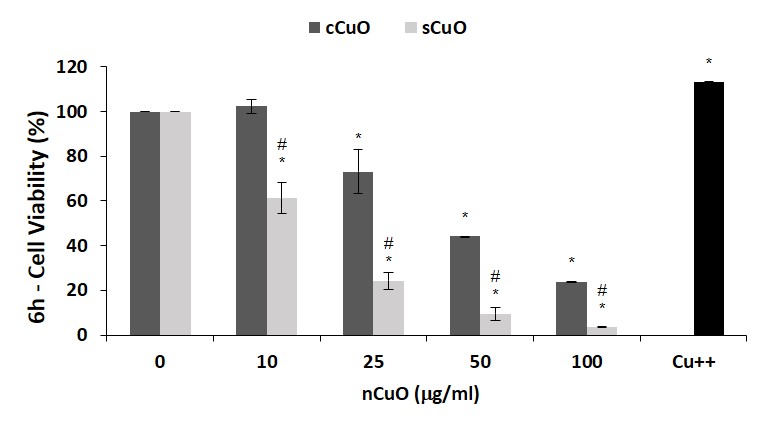


Supplementary Figure S9. Cell viability results by MTT assay of A549 cells exposed to CuO NPs (6 h). Dark grey bars = cCuO; light grey bars = sCuO; black bar= 80 μg/ml Cu^++^ (from CuSO_4_·5H_2_O). *Significantly different from control (ANOVA + Fisher LSD Method, p < 0,05). Differences among groups were considered statistically significant when p<0,05 by t-test (#).

**Intracellular ROS production**

Production of intracellular reactive oxygen species was assessed by DCFH_2_-DA reaction coupled with fluorescent microscopy detection.

Cells were seeded on glass coverslips placed in a 6 multi-well plate, at the density of 1,6 × 10^5^/well and after 24 h were preloaded with DCFH_2_-DA (30 min in the dark at 37°C, 5%, CO_2_ and 95% humidity). Cells were then washed twice with PBS and exposed for 1 h, in triplicate, to H_2_O_2_ 10 mM (as positive control), cCuO and sCuO at 25 and 50 µg/ml. At the end of the exposure time the coverslips were rinsed twice with PBS, incubated 20 min at RT with buffered formaldehyde 4% and rinsed twice again with PBS. After 5 minutes incubation with Hoechst (1:1000) they were mounted on microscope slides with Prolong antifade (Thermo-Fisher). Fluorescence was then detected by Axioplan Observer (Zeiss) Results from this experiment are summarised in Supplementary Fig. S10.


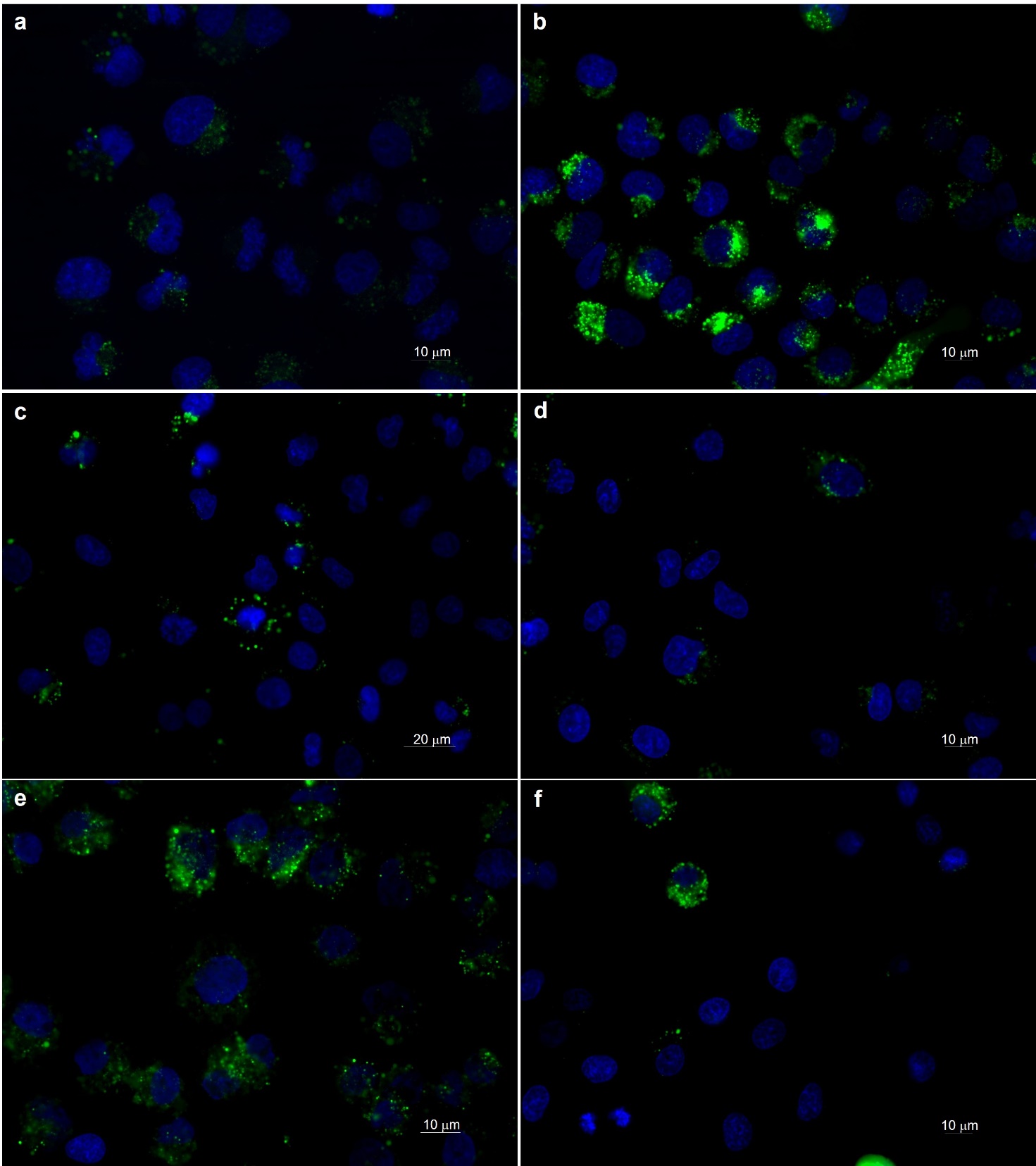


Supplementary Figure S10. Intracellular ROS production by DCFH_2_-DA in A549 exposed to nCuO for 1 h. (a) negative control (unexposed cells); (b) positive control (10 mM H_2_O_2_-exposed cells); (c) 25 μg/ml cCuO-exposed cells; (d) 50 μg/ml cCuO-exposed cells; (e) 25 μg/ml sCuO-exposed cells; (f) 50 μg/ml sCuO-exposed cells. Nuclei are stained by Hoechst (blue); ROS are shown as green spots.

**Co-localization ROS-mitochondria**

Co-localization of intracellular reactive oxygen species and mitochondria was assessed by DCFH_2_-DA reaction coupled with mitochondria staining by Mitotracker red.

Cells were seeded as described above. 30 minutes before exposure to CuO NPs A549 were preloaded with DCFH_2_-DA and Mitotracker red (30 min in the dark at 37°C, 5%, CO_2_ and 95% humidity). Cells were then washed twice with PBS and exposed for 1 h, in triplicate, to cCuO and sCuO at 50 µg/ml. At the end of the exposure time the coverslips were rinsed twice with PBS, incubated 20 min at RT with buffered formaldehyde 4% and rinsed twice again with PBS. After 5 minutes incubation with Hoechst (1:1000) they were mounted on microscope slides with Prolong antifade (Thermo-Fisher). Fluorescence was then detected by Axioplan Observer (Zeiss).

Results from this experiment are summarised in Supplementary Fig. S11.


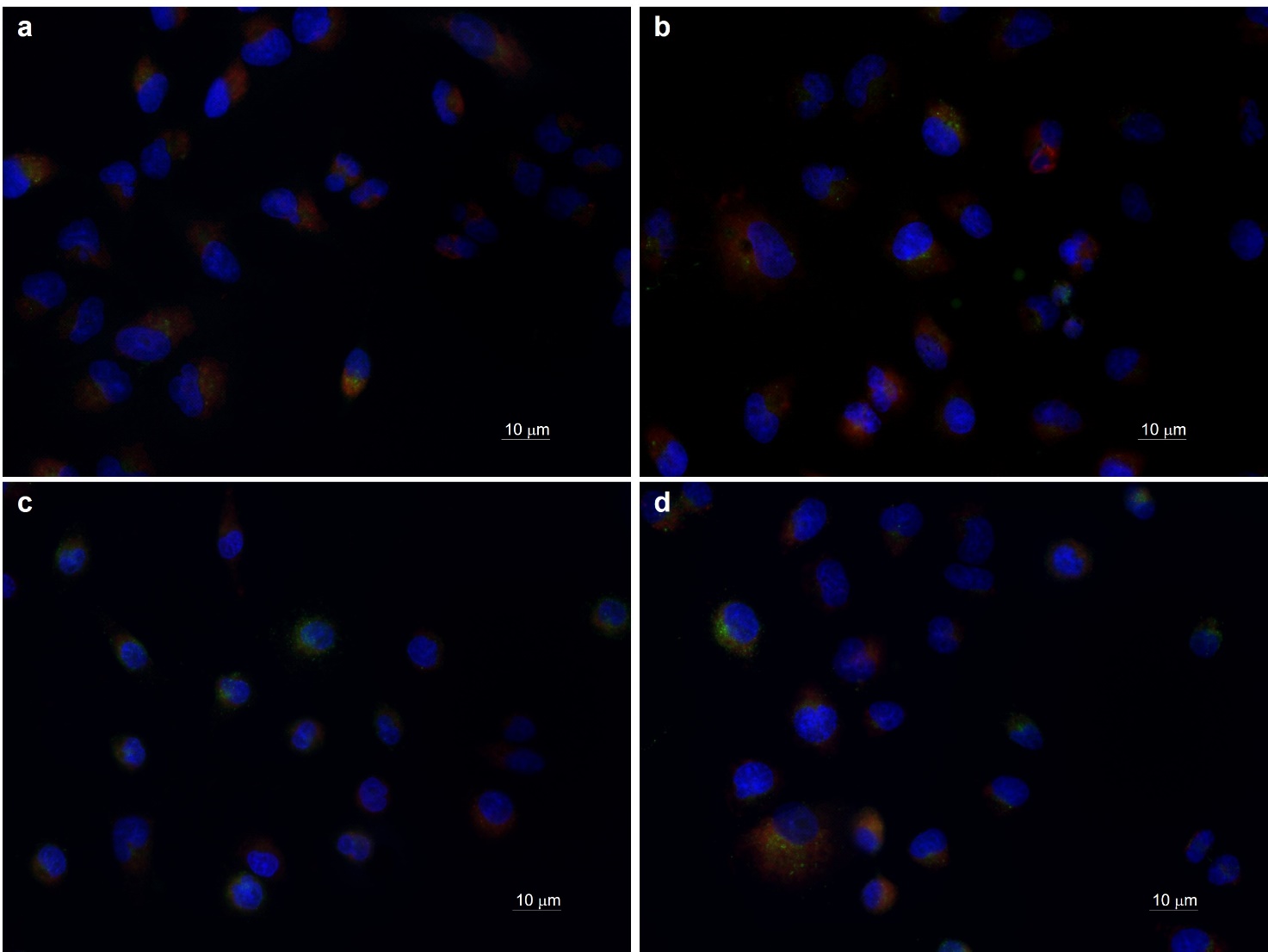


Supplementary Figure S11. Intracellular ROS-mitochondria co-localization in A549 exposed to nCuO for 1 h. (a) negative control (unexposed cells); (b) 50 μg/ml cCuO-exposed cells; (c) e (d) 50 μg/ml sCuO-exposed cells. Nuclei are stained by Hoechst (blue), ROS are shown as green spots, mitochondria are shown as red spots; co-localization is shown as orange-like areas.
